# Supplementary material for: Unveiling causal relationship between white matter tracts and psychiatric disorders
Source: Commun Biol. 2025 Aug 14;8:1221. doi: 10.1038/s42003-025-08669-6 (PMC12354733; doi:10.1038/s42003-025-08669-6)
Supplement: Supplementary file 1 — Supplementary materials [file 42003_2025_8669_MOESM1_ESM.pdf]

Causal Relationship between White Matter Tracts and Psychiatric Disorders: A Mendelian Randomization Study

Yifan Yu,[1] Tianye Jia,[2] Xiao Lin,[1] Yanping Bao,[3] Suhua Chang,[1] Jie Sun,[4] Teng Gao,[1] Jie Shi,[4, 6] Sizhi Ai,[6*] Kai Yuan,[1*]

1. Peking University Sixth Hospital, Peking University Institute of Mental Health, NHC Key Laboratory of Mental Health (Peking University), National Clinical Research Center for Mental Disorders (Peking University Sixth Hospital), Chinese Academy of Medical Sciences Research Unit (No.2018RU006), Peking University, Beijing, China.
2. Institute of Science and Technology for Brain-Inspired Intelligence, Fudan University, Shanghai, China.
3. National Institute on Drug Dependence and Beijing Key Laboratory of Drug Dependence, Peking University, Beijing 100191, China.
4. Center for Pain Medicine, Peking University Third Hospital, Beijing 100191, China.
5. Peking University Health Science Center, Peking University, Beijing, China.
6. Center for Sleep and Circadian Medicine, The Affiliated Brain Hospital of Guangzhou Medical University, Guangzhou, 510370, China.

* These authors jointly supervised this work

This file includes:

Supplementary Text
Figs. S1 to S25
Tables S1 to S2
Legends for Supplementary Data 1 - 23

Other Supplementary Materials for this manuscript include the following:

Supplementary Data 1 - 23

Contents

Preface 3
Supplementary Text 4
Mini-review of the association between WMTs and psychiatric disorders 4
ADHD and ASD 4
MDD, SCZ, BD, and PTSD 4
More details for forward Mendelian randomization 5
Fig. S1. Leave-one-out plot of ACR_MO on PTSD. 6
Fig. S2. Leave-one-out plot of CGH_MD on PTSD. 7
Fig. S3. Leave-one-out plot of CGH_RD on PTSD. 8
Fig. S4. Leave-one-out plot of FX_MO on ADHD. 9
Fig. S5. Leave-one-out plot of FXST_FA on SCZ. 10
Fig. S6. Leave-one-out plot of FXST_MO on BD. 11
Fig. S7. Leave-one-out plot of FXST_MO on TS. 12
Fig. S8. Leave-one-out plot of FXST_RD on PTSD. 13
Fig. S9. Leave-one-out plot of GCC_MD on MDD. 14
Fig. S10. Leave-one-out plot of GCC_MD on OUD. 15
Fig. S11. Leave-one-out plot of GCC_RD on OUD. 16
Fig. S12. Leave-one-out plot of IFO_AD on AUD. 17
Fig. S13. Leave-one-out plot of PCR_AD on PTSD. 18
Fig. S14. Leave-one-out plot of PCR_FA on PTSD. 19
Fig. S15. Leave-one-out plot of PCR_RD on PTSD. 20
Fig. S16. Leave-one-out plot of RLIC_MO on SCZ. 21
Fig. S17. Leave-one-out plot of RLIC_RD on PTSD. 22
Fig. S18. Leave-one-out plot of SCR_FA on SCZ. 23
Fig. S19. Leave-one-out plot of SFO_FA on PTSD. 24
Fig. S20. Leave-one-out plot of SFO_MD on PTSD. 25
Fig. S21. Leave-one-out plot of SLF_FA on PTSD. 26
Fig. S22. Leave-one-out plot of SLF_RD on PTSD. 27
Fig. S23. Leave-one-out plot of UNC_FA on PTSD. 28
Fig. S24. Leave-one-out plot of UNC_MD on PTSD. 29
Fig. S25. Scatter plot 30
Table S1. The summary of the association between WMTs and psychiatric disorders 31
Table S2. STROBE-MR checklist of recommended items to address in reports of Mendelian randomization studies [51,52] 41
References 45
Legends for Supplementary Data 1 to Supplementary Data 23 48

Preface

Due to the extensive use of abbreviations for white matter tracts (WMTs) and psychiatric disorders in this article, to avoid the disruption of readers' reading experience caused by the frequent appearance of abbreviations and their full forms, we have listed here the abbreviations for 21 WMTs and 10 psychiatric disorders presented in this article, for the convenience of readers.

Abbreviations of 21 WMTs:
ACR: anterior corona radiata
ALIC: anterior limb of internal capsule
BCC: body of corpus callosum
CGC: cingulum (cingulate gyrus)
CGH: cingulum (hippocampus)
CST: corticospinal tract
EC: external capsule
FX: fornix (column and body of fornix)
FXST: fornix (cres) / stria terminalis
GCC: genu of corpus callosum
IFO: inferior fronto-occipital fasciculus
PCR: posterior corona radiata
PLIC: posterior limb of the internal capsule
PTR: posterior thalamic radiation
RLIC: retrolenticular part of internal capsule
SCC: splenium of corpus callosum
SCR: superior corona radiata
SFO: superior fronto-occipital fasciculus
SLF: superior longitudinal fasciculus
SS: sagittal stratum
UNC: uncinate fasciculus

Abbreviations of 10 psychiatric disorders:
ADHD: attention deficit hyperactivity disorder
ASD: autism disorder
AUD: Alcohol use disorder
BD: bipolar disorder
CUD: cannabis use disorder
MDD: major depression disorder
PD: phobic anxiety disorders
PTSD: post-traumatic stress disorder
SCZ: schizophrenia
OUD: opioid use disorder

Abbreviations of 5 DTI parameters:
FA: fractional anisotropy
MD: mean diffusivities
AD: axial diffusivities
RD: radial diffusivities
MO: mode of anisotropy

Supplementary Text

Mini-review of the association between WMTs and psychiatric disorders

To obtain a brief overview of observational evidence regarding the association between WMTs and psychiatric disorders, we conducted a literature search on PubMed for articles published from August 2019 to August 2024. The keywords included "white matter tracts," "DTI," "Schizophrenia," "Bipolar disorder," "Cannabis use disorder," "Opioid use disorder," "Alcohol use disorder," "Post-traumatic stress disorder," "Major depressive disorder," "Autism disorder," "Tourette syndrome," and "Attention deficit hyperactivity disorder." Subsequently, we collected the observational findings of varying parameter changes in different WMTs across various psychiatric disorders. Finally, we got 868 results, and 32 articles were included (**Table S1**). Our review revealed that most current research focuses on the association between fractional anisotropy (FA) (32/32) values and psychiatric disorders. At the same time, discussions on mean diffusivity (MD) (7/32), axial diffusivity (AD) (3/32), and radial diffusivity (RD) (9/32) parameters were relatively limited. ADHD, ASD, SCZ, BD, MDD, and PTSD were the major psychiatric disorders researchers reported. Additionally, significant positive results exhibit specific regional dependencies: the limbic system, thalamic radiation, and corpus callosum, which correlate with their respective brain functions and clinical symptoms of the diseases. Although the association between WMTs and psychiatric disorders varies according to age, gender, and other factors, the preponderance of evidence currently supports that decreases in FA (28/32) and increases in MD (6/7), AD (2/3), and RD (7/9) were associated with the risk of psychiatric disorders and the severity of progression. The potential neurobiological mechanism may be as follows: alterations in the structure/function of WMTs change the efficiency or pattern of neuronal transmission within these tracts. The altered neuro signals originating from WMTs, upon processing by gray matter, produce abnormal neural decoding results, manifesting as neurological dysfunction. Overall, these studies qualitatively reflect the close association between WMTs and psychiatric disorders and reveal some of the associations between this association and tract type, as well as parameter type.

ADHD and ASD

The research on ADHD was controversial due to the different developing characteristics of white matter tracts in children. At the genetic level, children with higher polygenic risk scores (PRS) for ADHD tend to have more severe ADHD symptoms and a reduction of FA in several WMTs [1]. Furthermore, a sibling study revealed that AD values of some WMTs were significantly associated with ADHD symptoms: sustained attention and working memory [2]. Besides, the abnormalities in the anterior portion of the left inferior longitudinal fasciculus (ILF) can help us to distinguish between probands with persisting ADHD symptomatology and probands with desisting ADHD symptomatology [3]. However, a study illustrates that in-scanner head motion may fully mediate some causal mediation effects [4]. And some negative evidence showed no significant effect on FA in the ADHD children group(from a meta-analysis containing 15 studies, n= 607) [5] or for any specific tract [6].

Compared with ADHD, the changes in WMTs appear to be more pronounced in ASD. Post hoc analyses revealed significantly lower FA among children with ASD compared with children with ADHD. Furthermore, children with ASD had significantly higher mean MD, RD, and AD compared with those having ADHD [7]. Differences in white matter development between individuals whose ASD severity increased remained stable or decreased, suggesting that these functional differences were associated with fiber development in the autistic brain[8]. Autistic individuals whose autistic severity was increasing over time had a slower developmental trajectory of FA compared with individuals whose autistic severity was decreasing [8]. Moreover, a twin study of ASD indicated that genetic factors contributed to ~ 40% to 50% of the covariation between IQ scores and FA of the CC, while environmental factors contributed to ~ 10% to 20% of the covariation between ASD-related symptom severity and FA of the CP and EC, which means the WMTs alterations were associated with both genetic contributions and environmental influences[9]. We could find different alterations of WMTs in developmental disorders such as ADHD and ASD, but these changes did not seem to be very obvious (perhaps because the brain development of children is not complete, so it is not easy to observe obvious characteristics after covariate analysis with the control group).

MDD, SCZ, BD, and PTSD

The MDD population's complexity makes the research's influential factors more obvious. Reviewing 57 longitudinal studies investing the structural connectivity in MDD patients undergoing 4 different treatment methods: pharmacotherapy, psychotherapy, electroconvulsive therapy (ECT), and repetitive transcranial magnetic stimulation (rTMS), Tura et al. summarized that the results were widespread and inconsistent, which makes it difficult to make conclusions[10]. Gene polymorphism [11], negative stressful life events [12] , and social support were both related to WM integrity (lower FA values) in MDD. However, the results were robust when MDD patients had specific characteristics, such as acute episodes and non-suicidal self-injury (NSSI). MDD patients with NSSI showed reduced CG integrity compared to MDD patients without NSSI and healthy

controls[13]. And the dysfunction of the kynurenine/tryptophan ((Kyn/Trp) pathway may serve as a potential cause [14].

Compared with MDD, the alterations of WMTs in BD were more evident. BD patients showed higher Kyn levels and Kyn/Trp ratio than MDD patients and lower FA in several WM tracts, including the CC and the IFO. Lower Trp levels are associated with more severe depressive symptomatology irrespective of diagnosis and with lower FA in the CC and EC [15]. The present review summarizes studies on twin samples concordant or discordant for BD or MDD: WM integrity emerges as a hallmark of BD under genetic influence[16]. While the change of MDD only reached the trend level, BD showed a more significant decrease in FA, so disruptions in WMTs in BD might be a trait effect of the disorder[17]. Furthermore, there were different changes in FA in various subgroups of BD[18] , and better complex attention skills and executive functioning was associated with higher FA both globally and in the CC of BD patients [19].

The association between SCZ and WMTs is presented in detail in the **Table S1**. The psychopathological syndrome of formal thought disorder (FTD) is not only present in SCZ but also highly prevalent in MDD and BD [20]. Seitz-Holland J et al. observed evidence for cellular and extracellular white matter abnormalities in adolescent-onset psychosis (ADO). Although cellular white matter abnormalities were more prominent in ADO-SCZ, such alterations may reflect a shared trait, i.e., neurodevelopmental pathology, present across the psychosis spectrum. Extracellular abnormalities were evident in psychotic ADO-BD, potentially indicating a more dynamic, state-dependent brain reaction to psychosis [21]. 3 psychopathological formal thought disorder dimensions were delineated, i.e., disorganization, emptiness, and incoherence. Disorganization and incoherence were associated with global dysconnectivity[20].

From the perspective of WMTs deficits mechanism, MDD, and BD focused more on the Kyn/Trp pathway and inflammatory factors (proinflammatory and counter-regulatory immunomarkers, including regulatory T cells and natural killer cells markers) [22], while SCZ focuses on the response of cortisol [23].

For PTSD, in addition to the changes summarized in the **Table S1**, PTSD had some similarities with MDD: WMTs changes were more pronounced only in certain situations. The severity of PTSD symptom of those who experienced childhood maltreatment was inversely varied with FA in the IC [24]. Besides, there was evidence from shared genetics suggesting that PTSD was a subtype of MDD [25].

More details for forward Mendelian randomization
In forward MR, we identified causal associations for 24 significant WMT traits across 8 psychiatric disorders. (PTSD: 14, SCZ: 3, ADHD: 1, MDD: 1, OUD: 2, AUD: 1, BD: 1, TS: 1). The distribution of these WMTs includes FXST(4), GCC(3), PCR(3), CGH(2), RLIC(2), SFO(2), SLF(2), UNC(2), ACR(1), FX(1), IFO(1), SCR(1), which can be categorized into 5 groups: the thalamic radiations (thalamocortical, corticothalamic fibers)(11), limbic system(5), connections between left/right cerebral hemisphere or different lobes(6), and connections between the cortex(2). The parameters of WMTs encompass MO(5), FA(6), RD(6), AD(2), and MD(5), reflecting 4 presumed types of structural damage: axonal damage (indicated by AD↑), cellular membrane damage (indicated by MD↑), myelin damage (indicated by RD↑), and white matter tract integrity damage (indicated by FA↓). Among the 24 WMT-psychiatric disorder pairs, 12 unique WMTs were identified. In addition to PTSD being widely distributed, the significant causal associations of 7 other psychiatric disorders were concentrated in 6 WMTs.

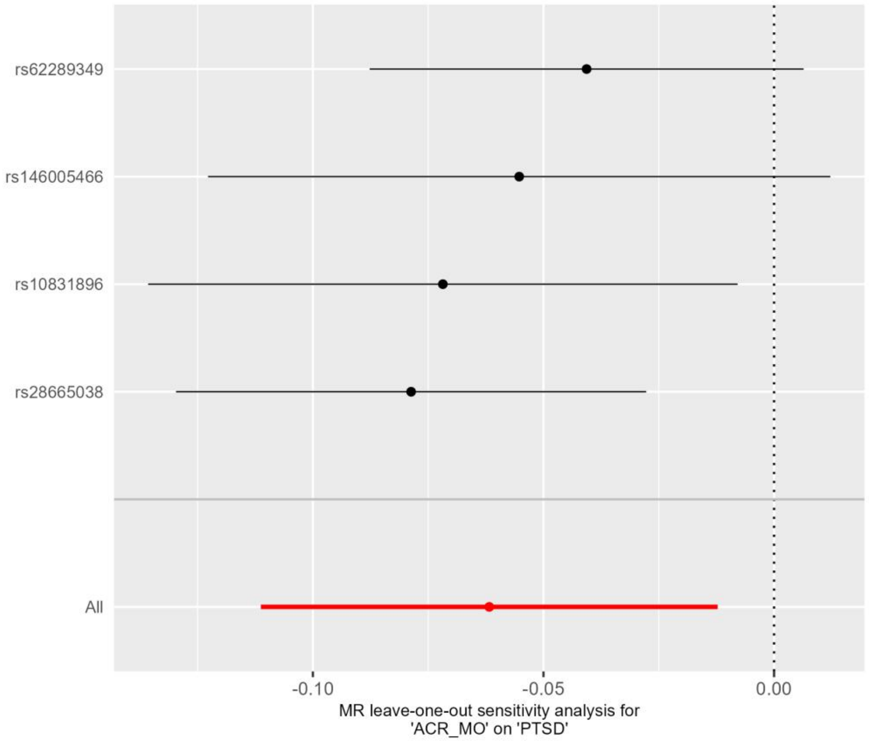

Fig. S1. Leave-one-out plot of ACR_MO on PTSD.

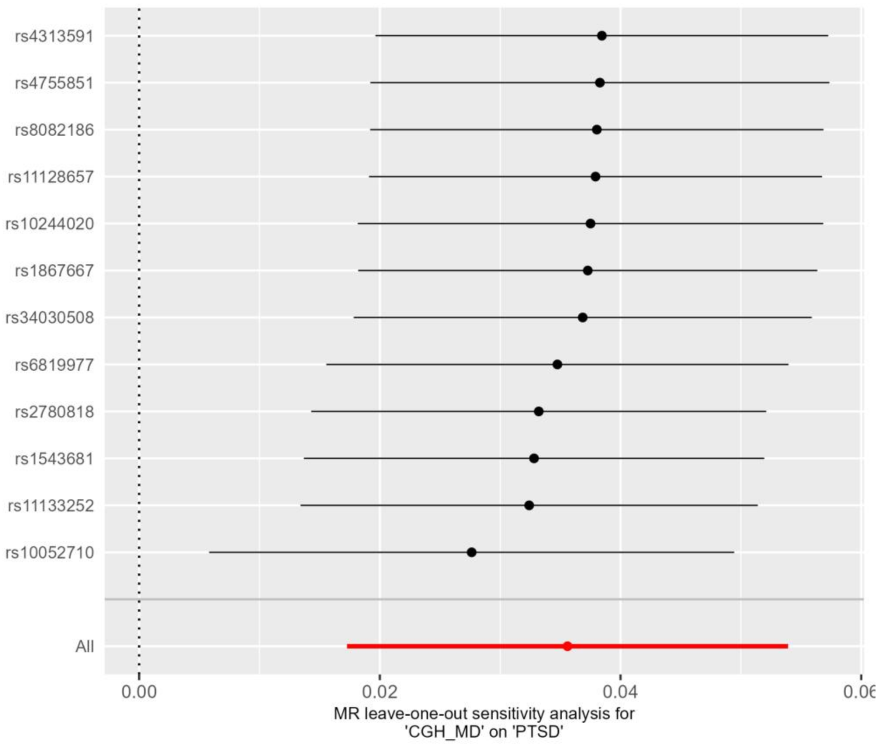

Fig. S2. Leave-one-out plot of CGH_MD on PTSD.

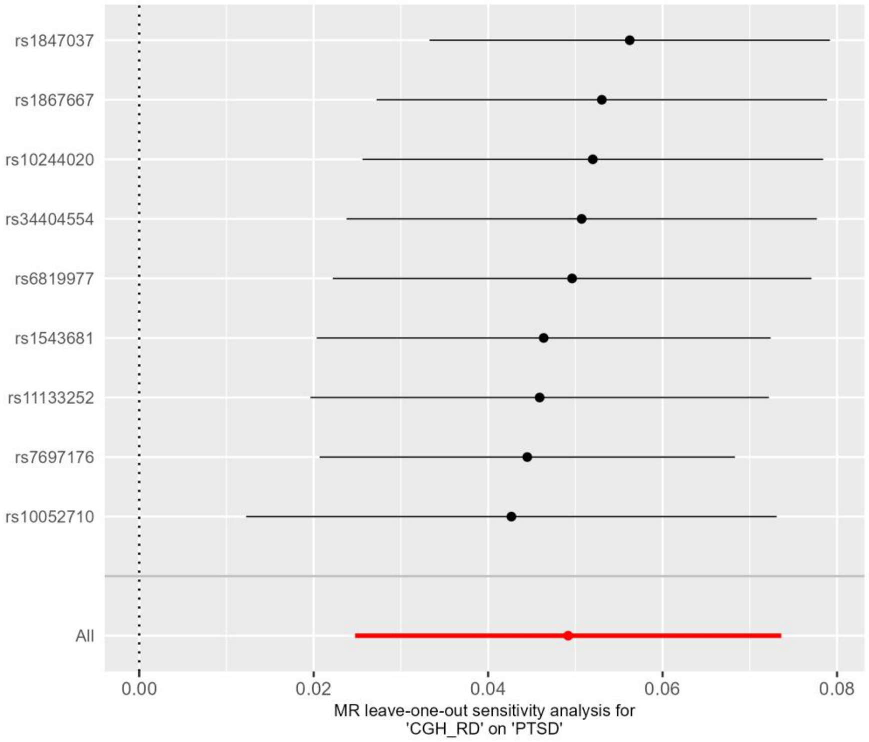

Fig. S3. Leave-one-out plot of CGH_RD on PTSD.

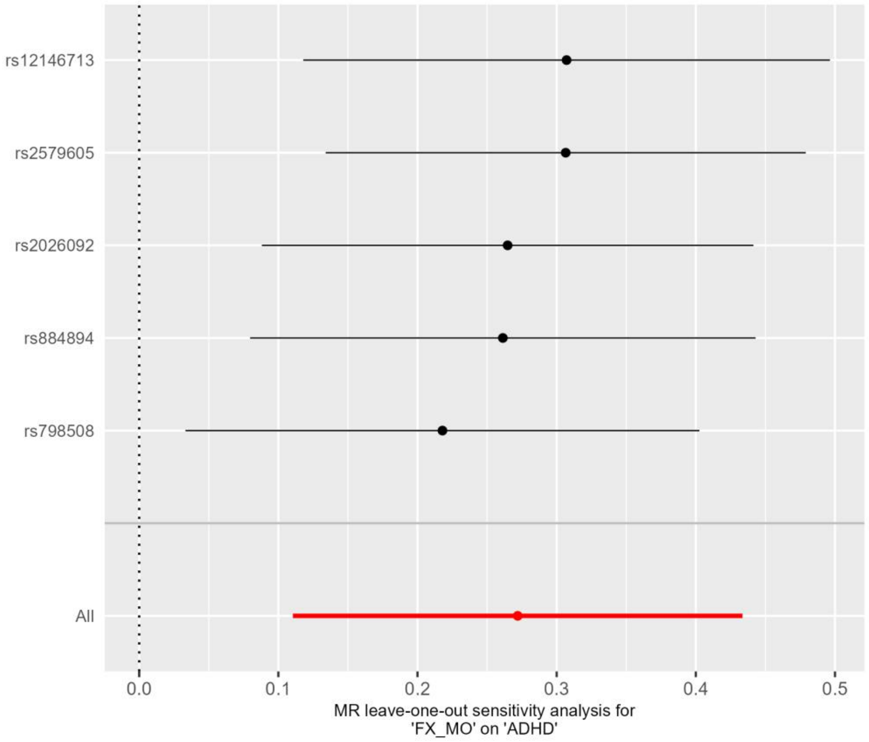

Fig. S4. Leave-one-out plot of FX_MO on ADHD.

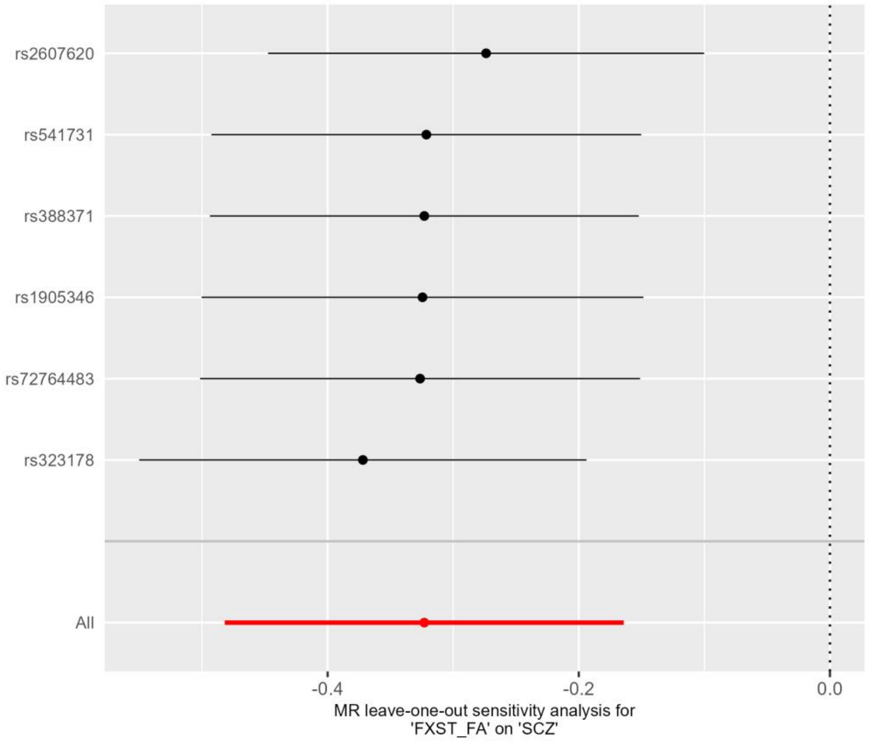

Fig. S5. Leave-one-out plot of FXST_FA on SCZ.

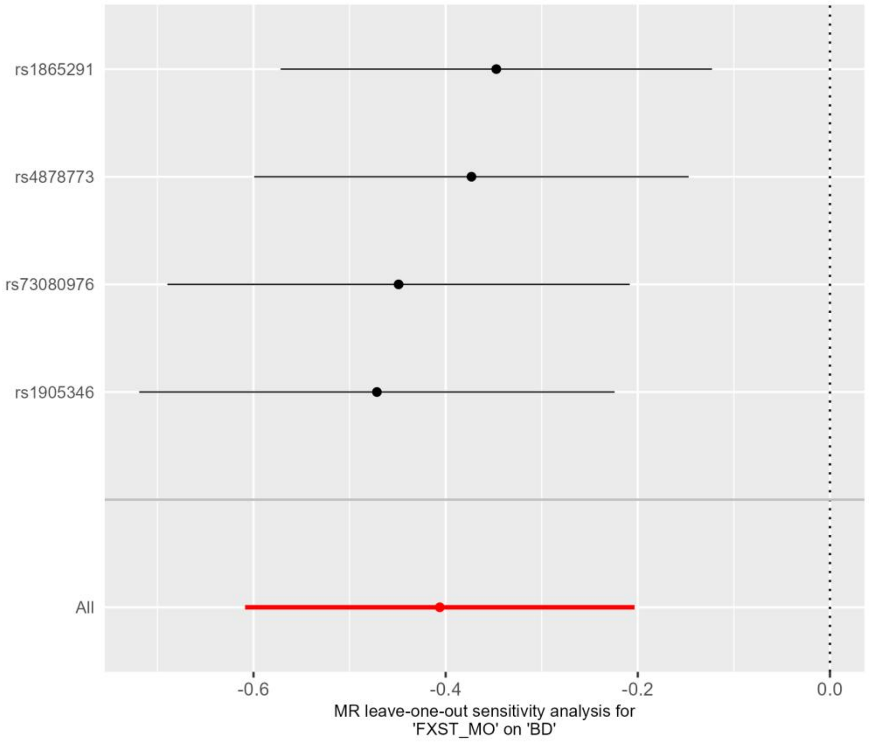

Fig. S6. Leave-one-out plot of FXST_MO on BD.

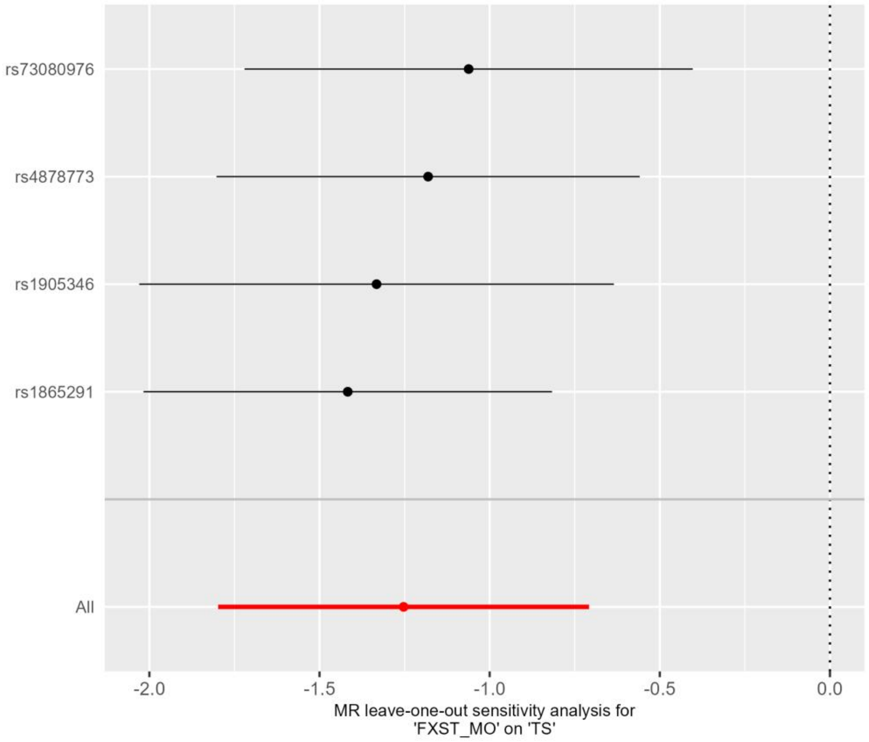

Fig. S7. Leave-one-out plot of FXST_MO on TS.

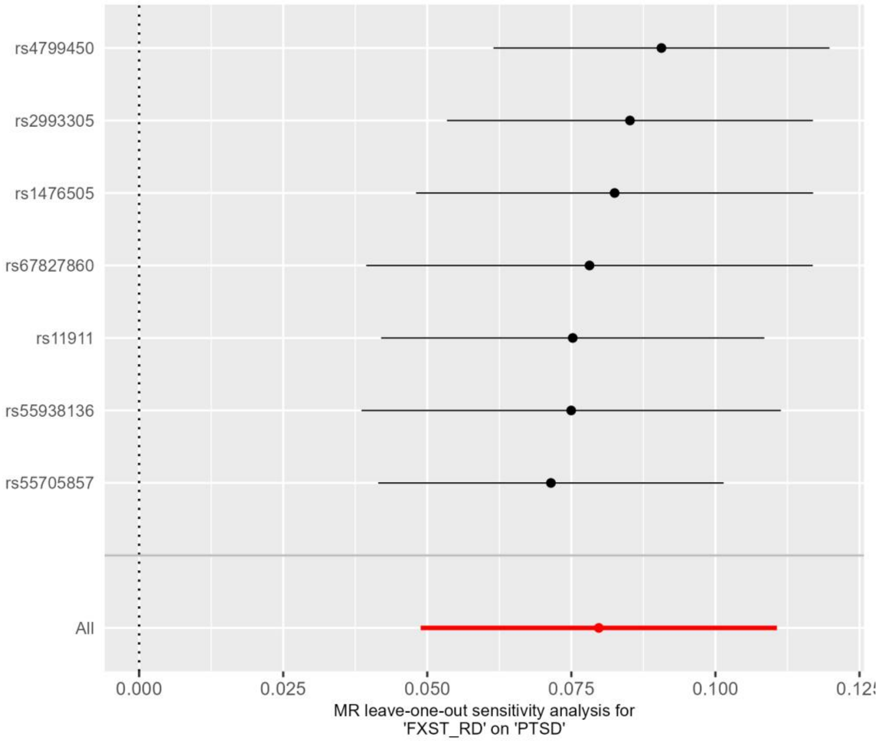

Fig. S8. Leave-one-out plot of FXST_RD on PTSD.

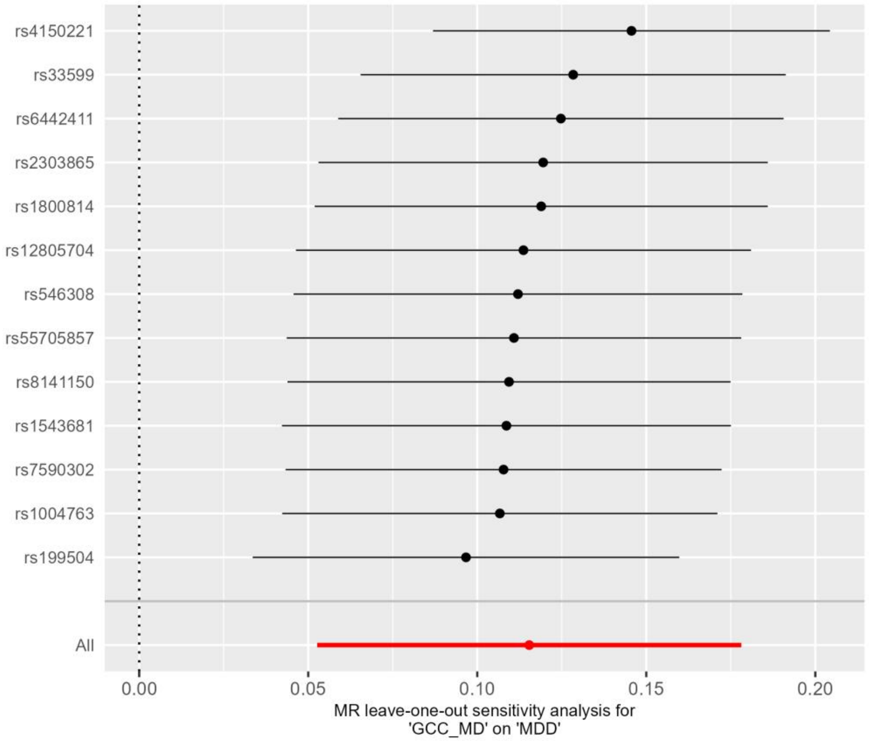

Fig. S9. Leave-one-out plot of GCC_MD on MDD.

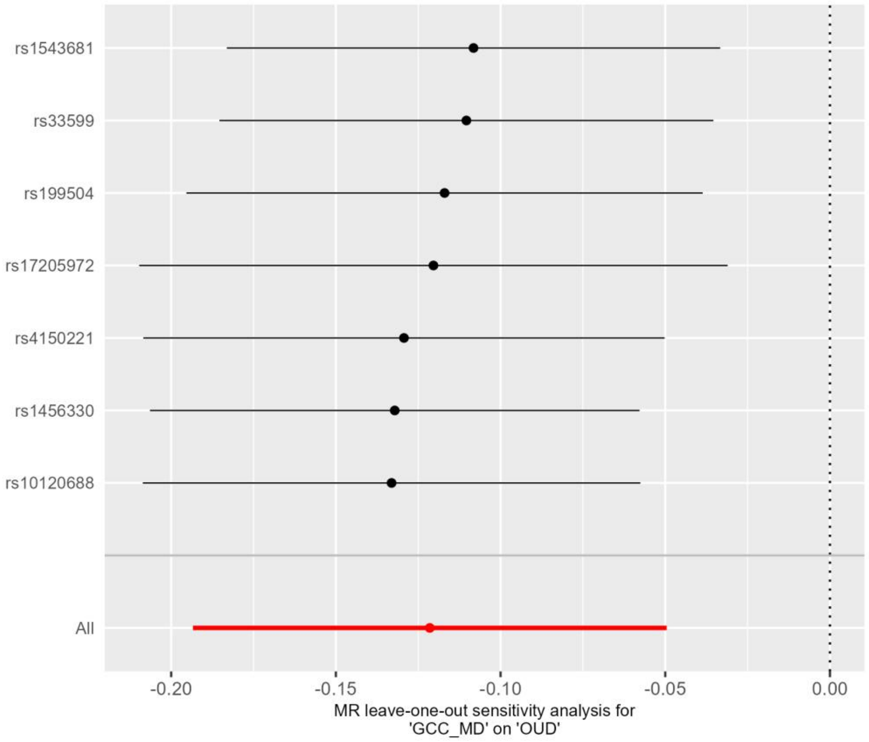

Fig. S10. Leave-one-out plot of GCC_MD on OUD.

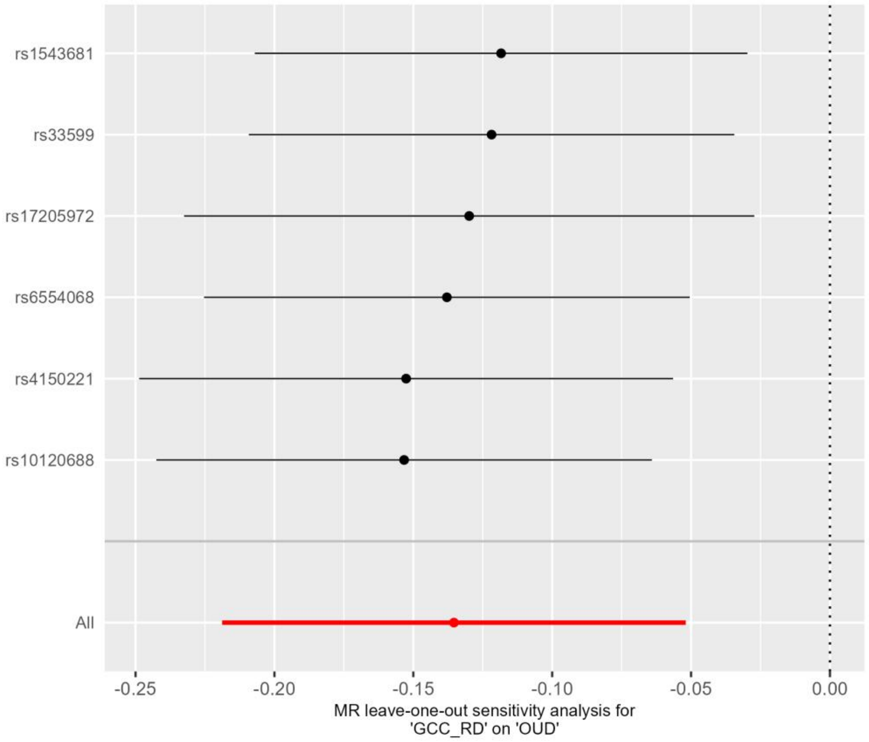

Fig. S11. Leave-one-out plot of GCC_RD on OUD.

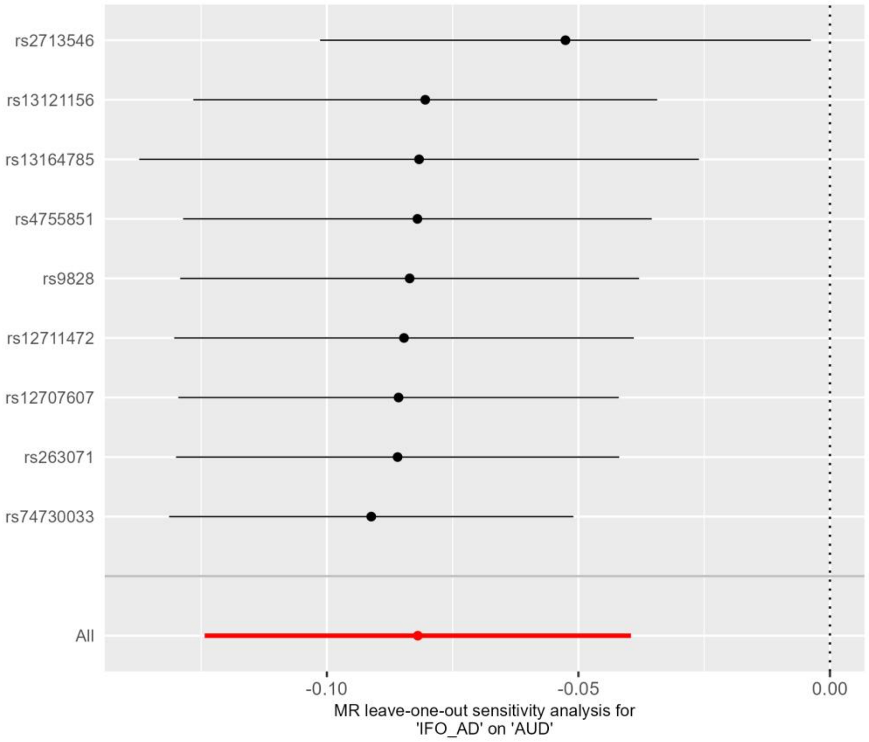

Fig. S12. Leave-one-out plot of IFO_AD on AUD.

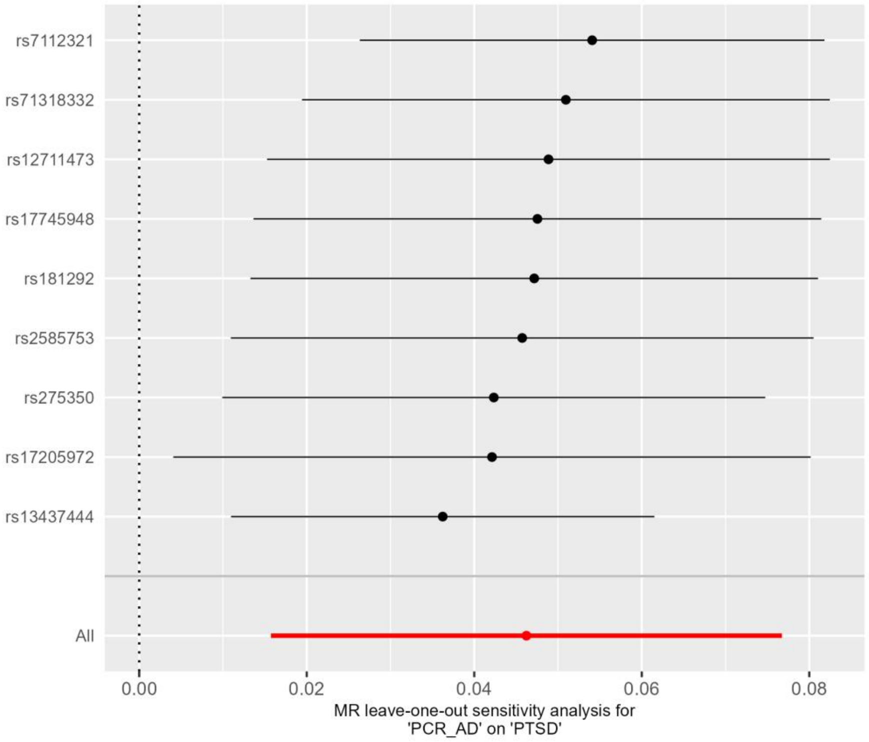

Fig. S13. Leave-one-out plot of PCR_AD on PTSD.

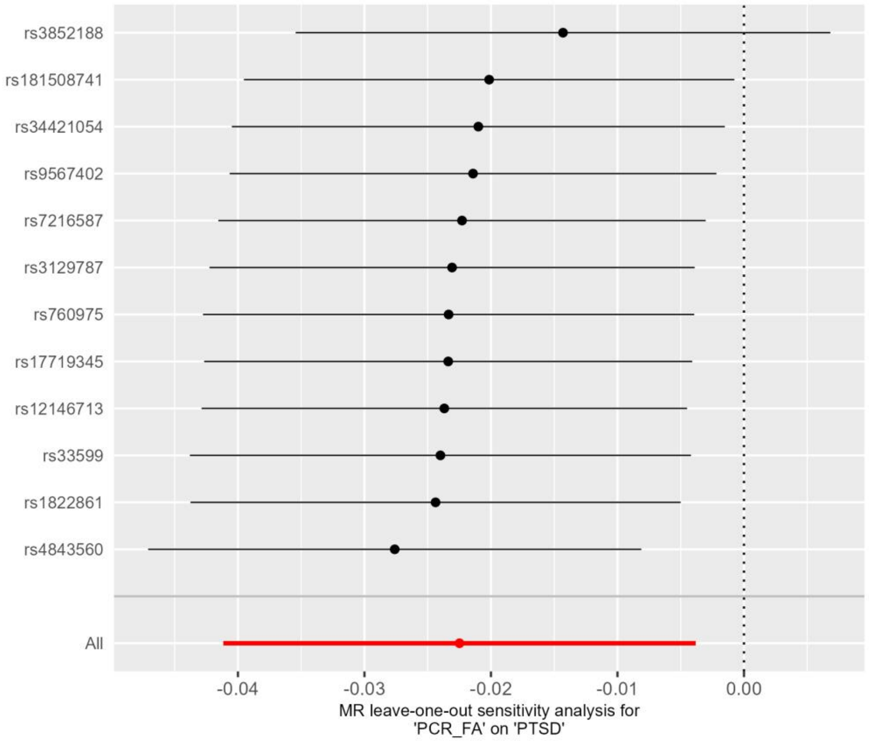

Fig. S14. Leave-one-out plot of PCR_FA on PTSD.

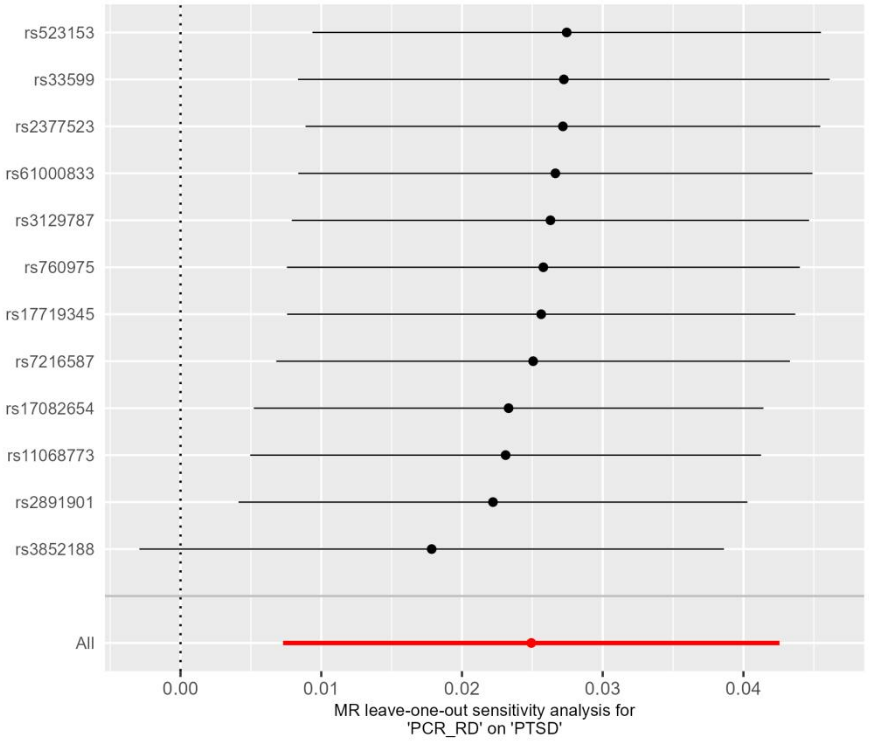

Fig. S15. Leave-one-out plot of PCR_RD on PTSD.

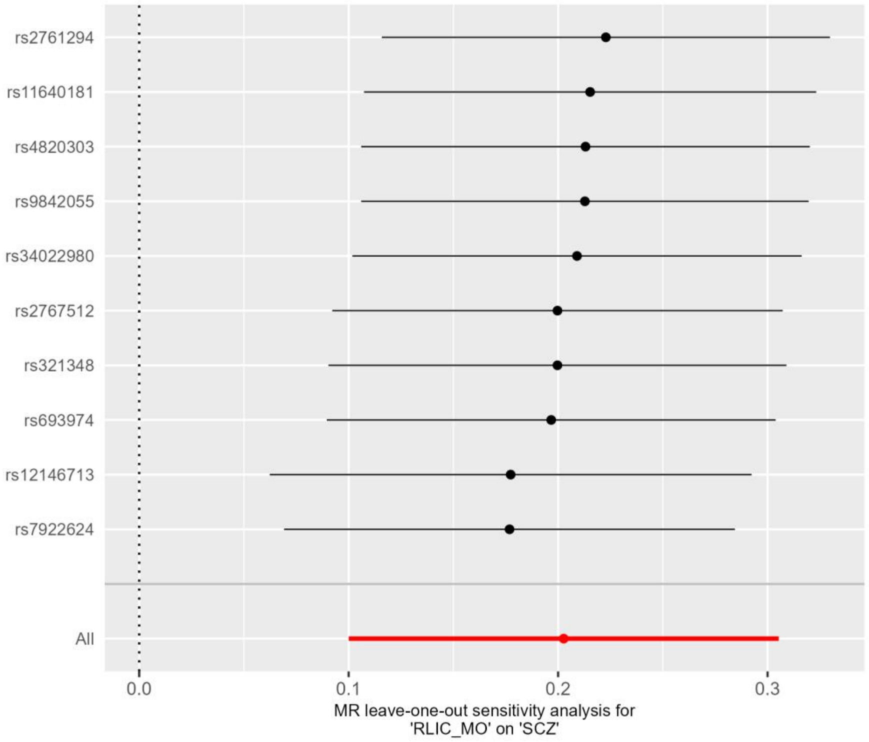

Fig. S16. Leave-one-out plot of RLIC_MO on SCZ.

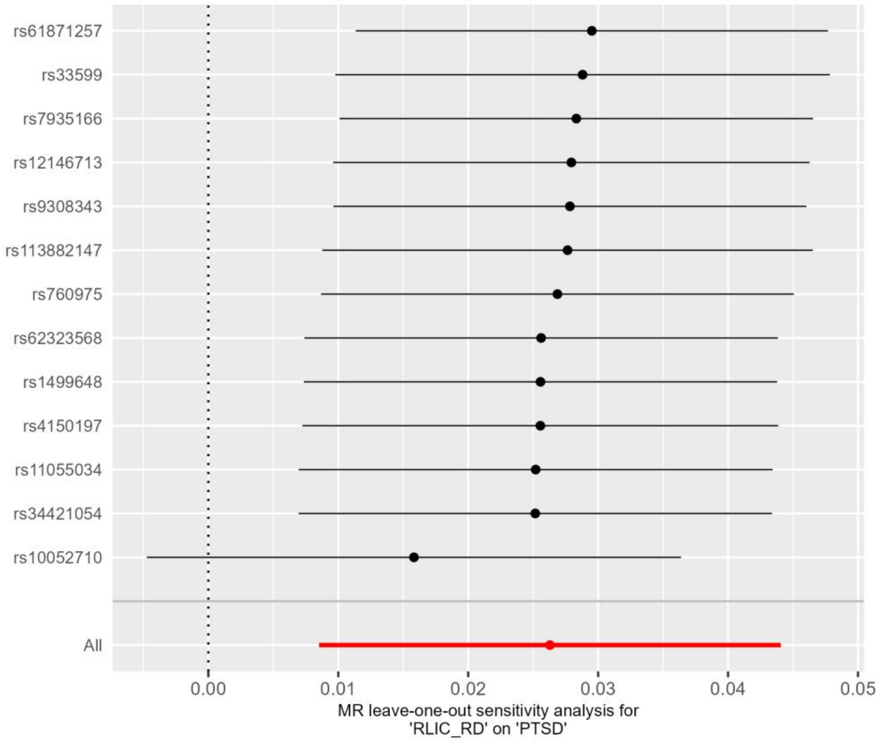

Fig. S17. Leave-one-out plot of RLIC_RD on PTSD.

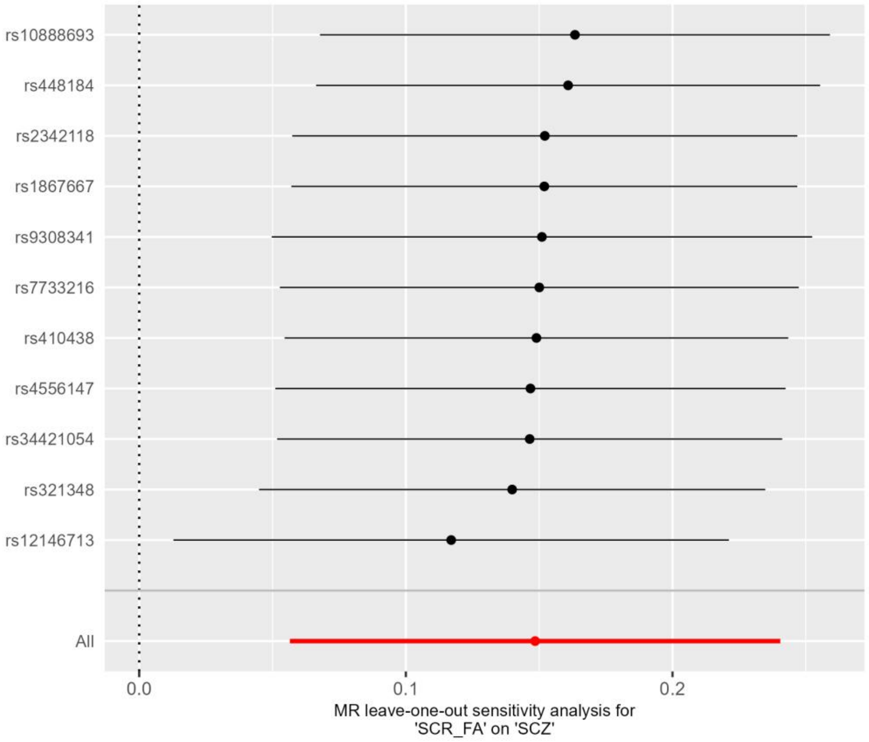

Fig. S18. Leave-one-out plot of SCR_FA on SCZ.

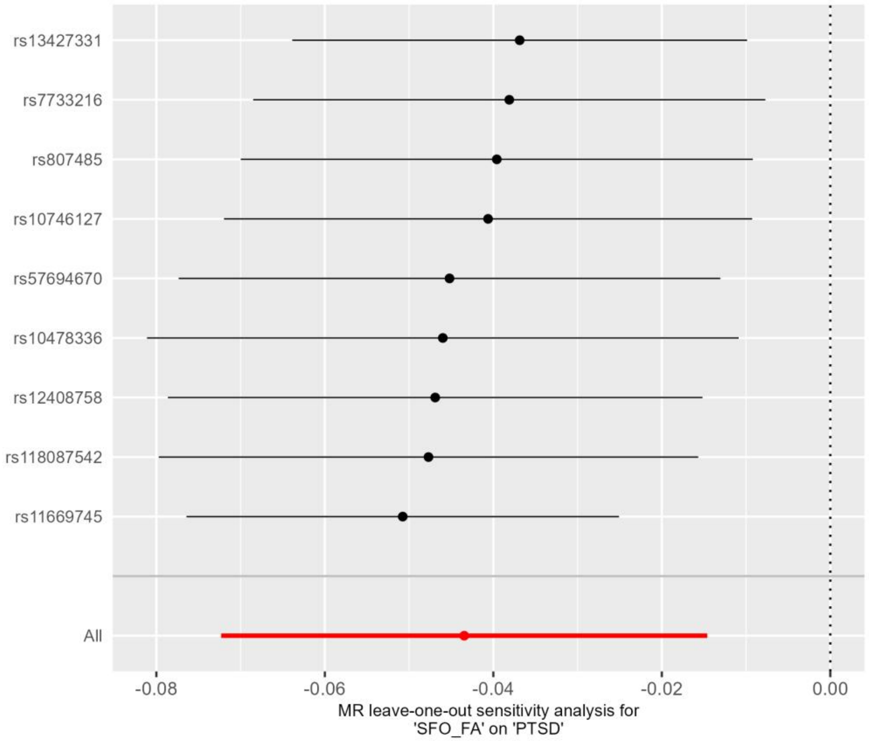

Fig. S19. Leave-one-out plot of SFO_FA on PTSD.

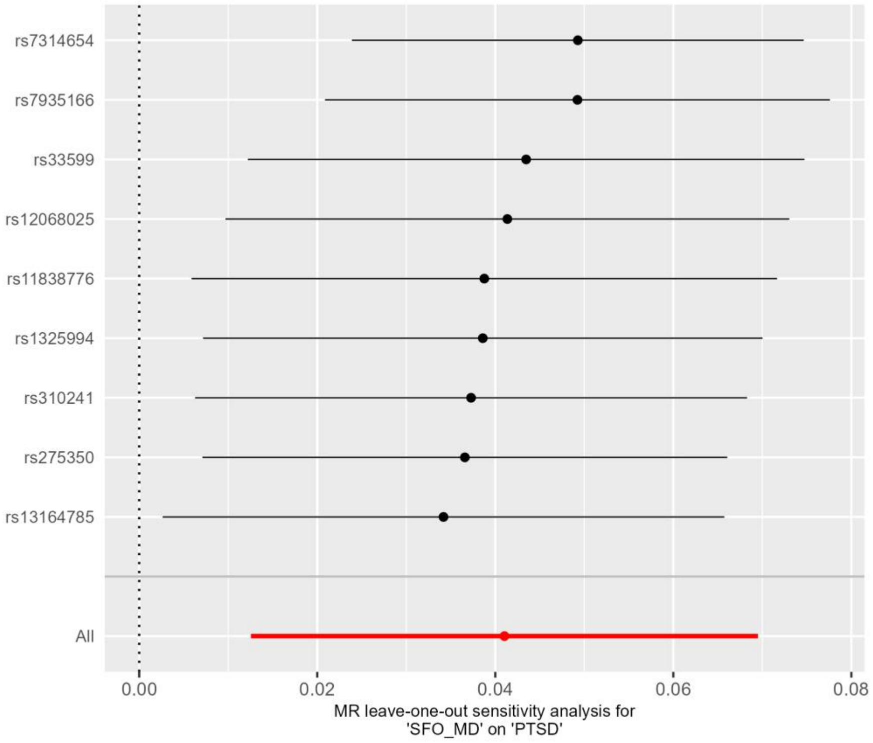

Fig. S20. Leave-one-out plot of SFO_MD on PTSD.

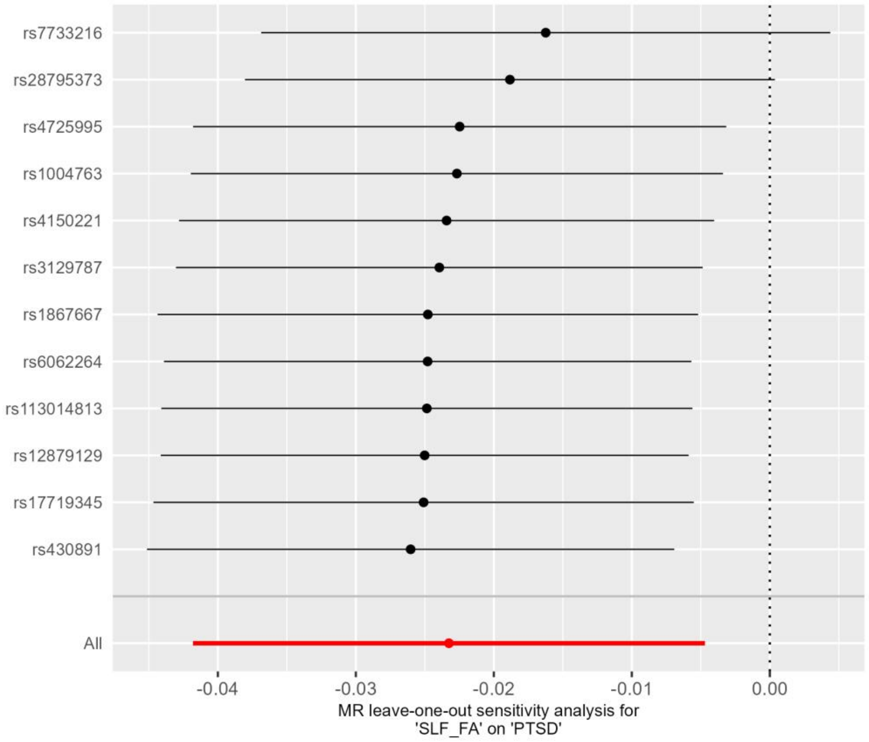

Fig. S21. Leave-one-out plot of SLF_FA on PTSD.

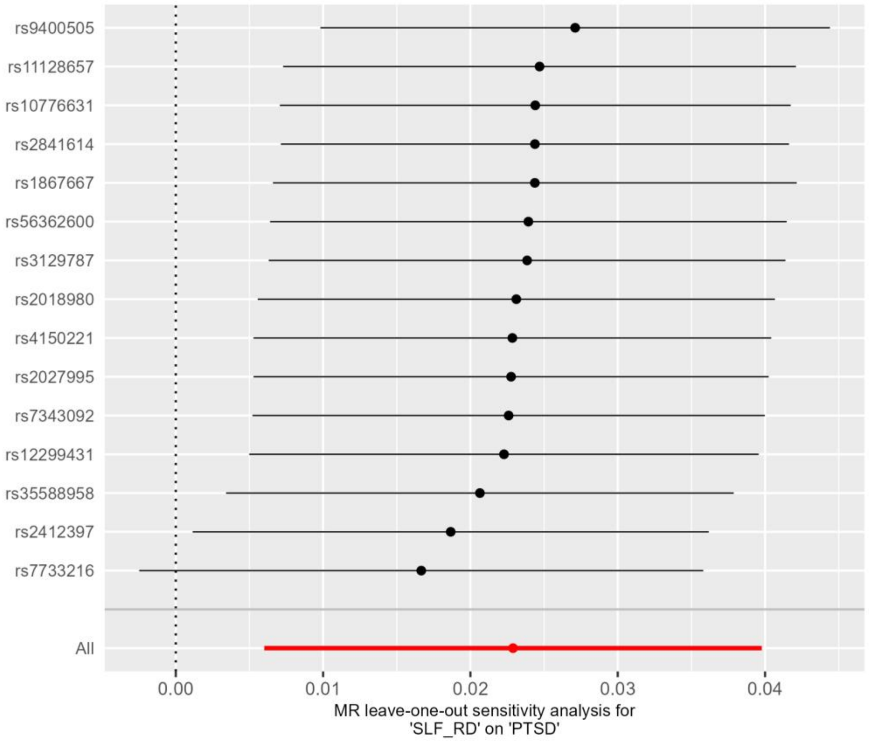

Fig. S22. Leave-one-out plot of SLF_RD on PTSD.

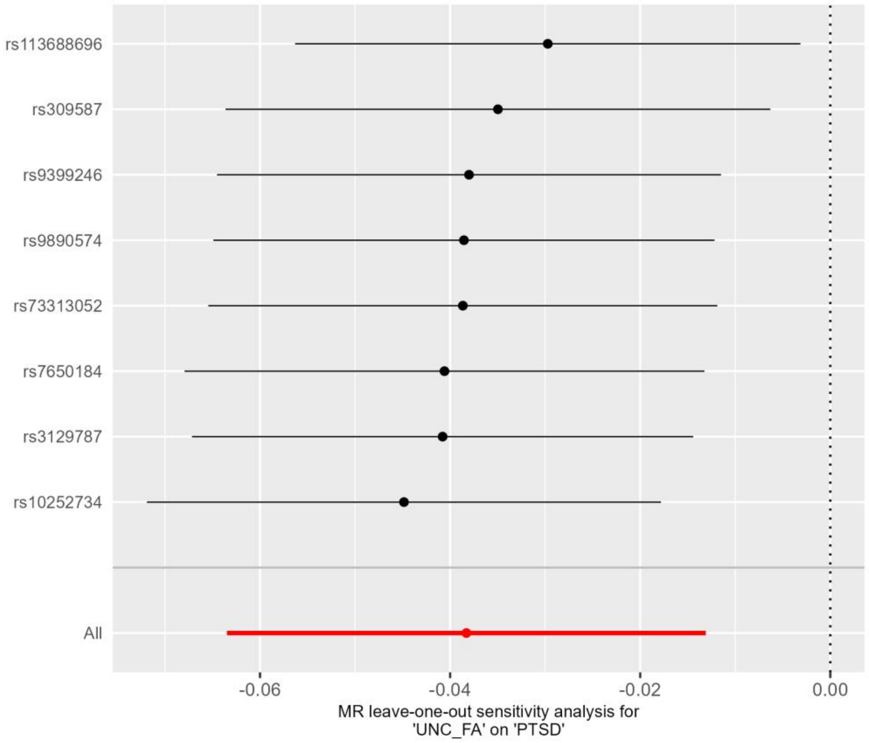

Fig. S23. Leave-one-out plot of UNC_FA on PTSD.

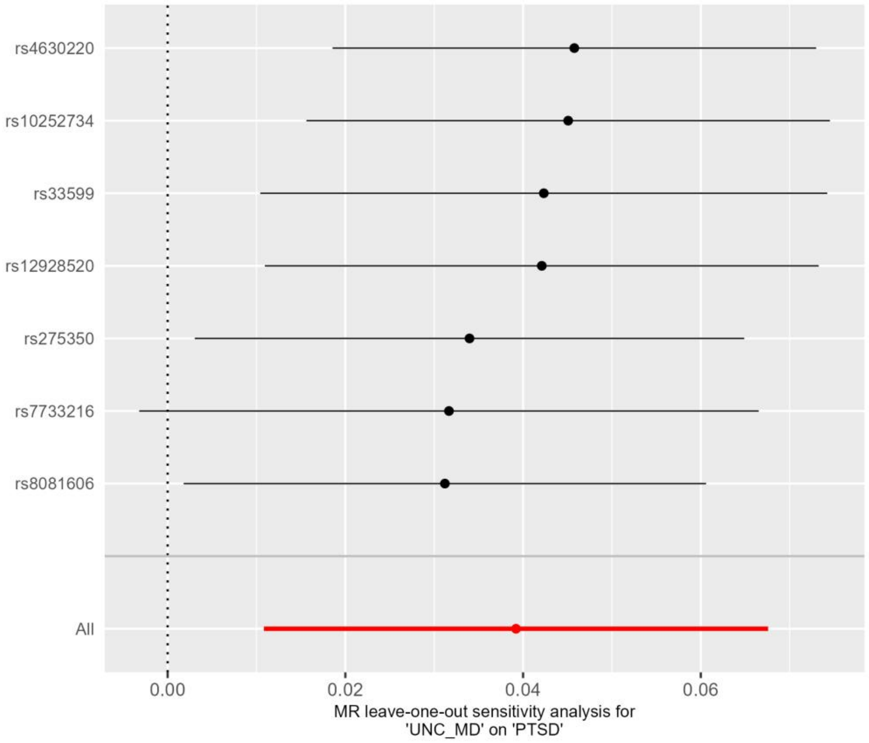

Fig. S24. Leave-one-out plot of UNC_MD on PTSD.

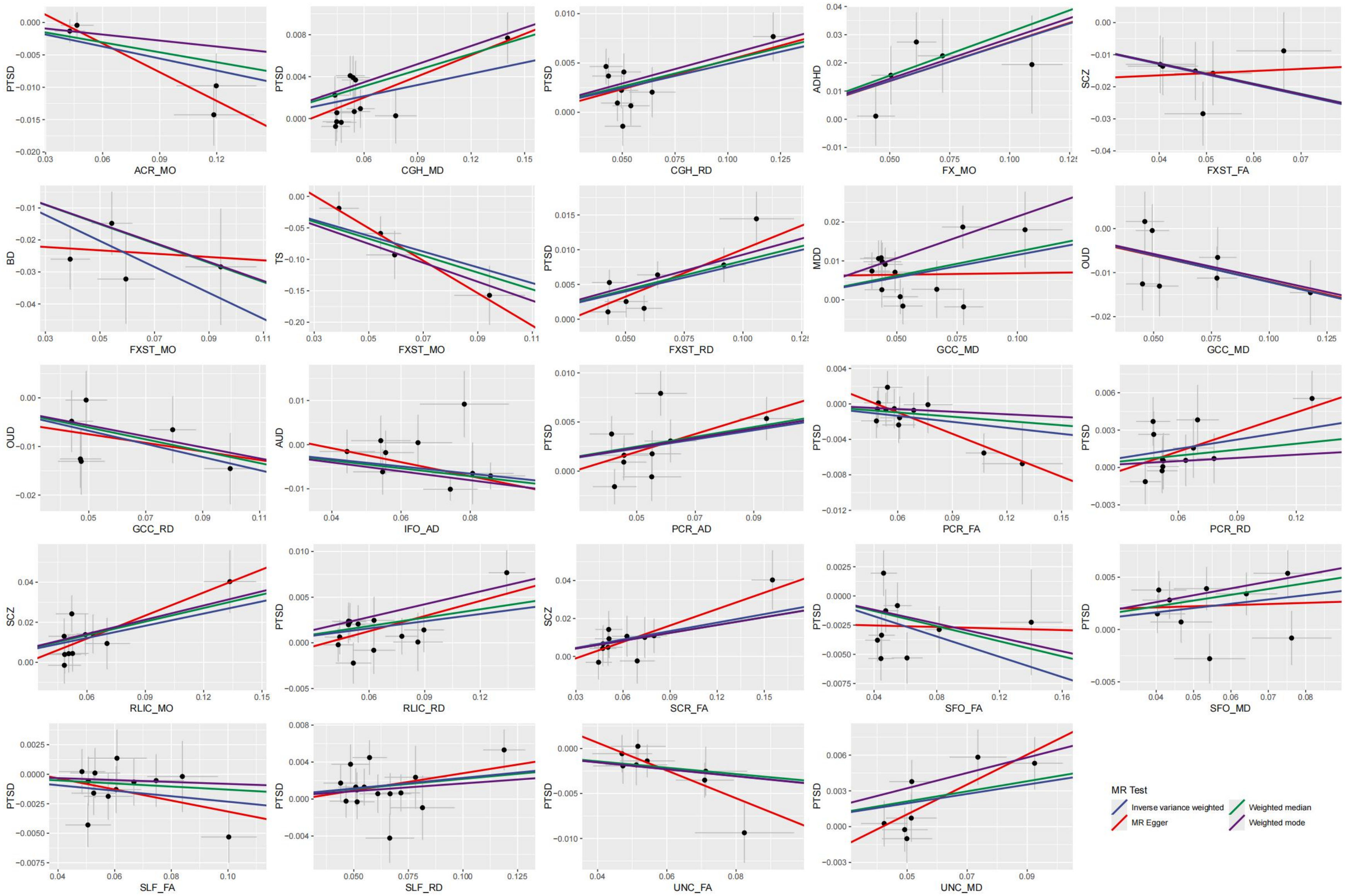

Fig. S25. Scatter plot

Table S1. The summary of the association between WMTs and psychiatric disorders

White matter tracts	Psychiatric disorders	Parameters	Alteration	Patient	Control	Study design	Note and Reference
CC	ADHD	AD	↑	50	50	Sibling design, RCT	Unaffected siblings were in the intermediate position between those of the ADHD and control groups. [2]
CST	ADHD	AD	↑	50	50	Sibling design, RCT	Unaffected siblings were in the intermediate position between those of the ADHD and control groups. [2]
Perpendicular fasciculus	ADHD	AD	↑	50	50	Sibling design, RCT	Unaffected siblings were in the intermediate position between those of the ADHD and control groups. [2]
SLF	ADHD	AD	↑	50	50	Sibling design, RCT	Unaffected siblings were in the intermediate position between those of the ADHD and control groups. [2]
BCC	BD	AD	↑	211	1506	Case-control	[26]
FX	BD	AD	↑	211	1506	Case-control	[26]
PLIC	BD	AD	↑	211	1506	Case-control	[26]
FX	SCZ	AD	↑	696	1506	Case-control	[26]
PCR	SCZ	AD	↑	696	1506	Case-control	[26]
SCR	SCZ	AD	↑	696	1506	Case-control	[26]
UNC	SCZ	AD	↑	696	1506	Case-control	[26]
GCC	BD	AD	↓	36	38	Case-control	[27]
PLIC	BD	AD	↓	36	38	Case-control	[27]
SCR	BD	AD	↓	36	38	Case-control	[27]
BCC	ADHD	FA	↑	278 in total	278 in total	Meta 6 studies	[5]
Fronto-straital tract	ADHD	FA	↑	50	50	Sibling design, RCT	Unaffected siblings were in the intermediate position between those of the ADHD and control groups. [2]
Precuneus	ADHD	FA	↑	278 in total	278 in total	Meta 6 studies	[5]
SLF	ADHD	FA	↑	278 in total	278 in total	Meta 6 studies	[5]
Cerebellum	ASD	FA	↑	117 in total	117 in total	Meta 6 studies	[5]
CST	ASD	FA	↑	117 in total	117 in total	Meta 6 studies	[5]
SLF	ASD	FA	↑	278 in total	278 in total	Meta 6 studies	[5]

IFO	PTSD	FA	↑	80	103	Meta<br>3 studies	[28]
ALIC	SCZ	FA	↑	1543	983	Meta	SCZ vs BD [29]
BCC	ADHD	FA	↓	-	-	Meta<br>17 studies	[30]
BCC	ADHD	FA	↓	278 in total	278 in total	Meta<br>6 studies	[5]
CC	ADHD	FA	↓	55	50	Case-control	[7]
Cerebellum	ADHD	FA	↓	-	-	Meta<br>8 studies	[30]
MCP	ADHD	FA	↓	-	-	Meta<br>8 studies	[30]
CG	ADHD	FA	↓	-	-	Meta<br>21 studies	[30]
CG	ADHD	FA	↓	128	58	Case-control	[3]
Thalamus and the ATR	ADHD	FA	↓	-	-	Meta<br>32 studies	[30]
GCC	ADHD	FA	↓	278 in total	278 in total	Meta<br>6 studies	[5]
IFO	ADHD	FA	↓	-	-	Meta<br>11 studies	[30]
ILF	ADHD	FA	↓	-	-	Meta<br>13 studies	[30]
ILF	ADHD	FA	↓	128	58	Case-control	Only abnormalities in the anterior portion of the left ILF distinguished probands with persisting versus desisting ADHD symptomatology, suggesting that abnormalities in the cingulum angular bundle might reflect "scarring" effects of childhood ADHD. [3]
ILF	ADHD	FA	↓	951	4884	Meta<br>5 cohorts	[31]
ICP	ADHD	FA	↓	16	20	Case-control	[32]

PILC/CST	ADHD	FA	↓	-	-	Meta<br>15 studies	[30]
SCC	ADHD	FA	↓	-	-	Meta<br>17 studies	[30]
SCC	ADHD	FA	↓	278 in total	278 in total	Meta<br>6 studies	[5]
SLF	ADHD	FA	↓	-	-	Meta<br>17 studies	[30]
UNF	ADHD	FA	↓	951	4884	Meta<br>5 cohorts	[31]
BCC	ASD	FA	↓	126	1506	Case-control	[26]
BCC	ASD	FA	↓	176 in total	176 in total	Meta<br>6 studies	[5]
BCC	ASD	FA	↓	176 in total	176 in total	Meta<br>6 studies	[5]
BCC	ASD	FA	↓	26	26	Cross-sectional	[33]
CC	ASD	FA	↓	69	50	Case-control	[7]
CG	ASD	FA	↓	125	69	Longitudinal cohort	[8]
Cortico-basal ganglia tract	ASD	FA	↓	49	96	Case-control	[34]
Arcuate fasciculus/SLF	ASD	FA	↓	596	587	Meta<br>26 studies	Dorsal pathway: language-related.[66]
Forceps major	ASD	FA	↓	26	26	Cross-sectional	[33]
Forceps minor	ASD	FA	↓	26	26	Cross-sectional	[33]
GCC	ASD	FA	↓	278 in total	278 in total	Meta<br>6 studies	[5]
IC	ASD	FA	↓	125	69	Longitudinal cohort	[8]
IFO	ASD	FA	↓	-	-	Meta<br>9 studies	[35]
IFOF	ASD	FA	↓	26	26	Cross-sectional	[33]
ILF	ASD	FA	↓	278 in total	278 in total	Meta<br>10 studies	[35]
SCC	ASD	FA	↓	278 in total	278 in total	Meta<br>6 studies	[5]
SCC	ASD	FA	↓	125	69	Longitudinal cohort	[8]
SLF	ASD	FA	↓	26	26	Cross-sectional	[33]
SLF	ASD	FA	↓	125	69	Longitudinal cohort	[8]

SS	ASD	FA	↓	125	69	Longitudinal cohort	[8]
Thalamus	ASD	FA	↓	117 in total	117 in total	Meta 6 studies	[5]
UNC	ASD	FA	↓	316	321	Meta 12 studies	[35]
UNC	ASD	FA	↓	125	69	Case-control	[8]
ACR	BD	FA	↓	49	32	Case-control	[36]
BCC	BD	FA	↓	2054	2577	Meta 57 studies	[37]
CC	BD	FA	↓	1482	1551	Meta 26 cohorts	[38]
CC	BD	FA	↓	983	1612	Case-control	[29]
CG	BD	FA	↓	1482	1551	Meta 26 cohorts	[38]
CGC	BD	FA	↓	211	1506	Case-control	[26]
CST	BD	FA	↓	49	32	Case-control	[36]
CST	BD	FA	↓	36	38	Case-control	[27]
Forceps major	BD	FA	↓	136	136	Case-control	[17]
Forceps minor	BD	FA	↓	136	136	Case-control	[17]
Fronto-orbito-polar tracts	BD	FA	↓	-	-	Meta 57 studies	This fiber is related to reward processing. [37]
GCC	BD	FA	↓	36	38	Case-control	[27]
IFO	BD	FA	↓	136	136	Case-control	[17]
ILF	BD	FA	↓	136	136	Case-control	[17]
PCR	BD	FA	↓	49	32	Case-control	[36]
PLIC	BD	FA	↓	49	32	Case-control	[36]
SCC	BD	FA	↓	-	-	Meta 57 studies	[37]
SCC	BD	FA	↓	36	38	Case-control	[27]
SCR	BD	FA	↓	49	32	Case-control	[36]
SCR	BD	FA	↓	36	38	Case-control	[27]
SLF	BD	FA	↓	2054	2577	Case-control	[37]
SLF	BD	FA	↓	136	136	Case-control	[17]

SLF	BD	FA	↓	36	38	Case-control	[27]
ATR	BD	FA	↓	2054	2577	Case-control	This fiber is related to reward processing. [37]
Forceps minor	Depression	FA	↓	4471	4182	Case-control	[39]
TR	Depression	FA	↓	4471	4182	Case-control	[39]
ALIC	MDD	FA	↓	-	-	Meta 84 studies	[40]
BCC	MDD	FA	↓	-	-	Meta 84 studies	[40]
CG	MDD	FA	↓	-	-	Meta 84 studies	[40]
ACR	MDD	FA	↓	-	-	Meta 84 studies	[40]
SCR	MDD	FA	↓	-	-	Meta 84 studies	[40]
PCR	MDD	FA	↓	-	-	Meta 84 studies	[40]
GCC	MDD	FA	↓	-	-	Meta 84 studies	[40]
IFO	MDD	FA	↓	-	-	Meta 84 studies	[40]
ILF	MDD	FA	↓	-	-	Meta 84 studies	[40]
SCC	MDD	FA	↓	-	-	Meta 84 studies	[40]
SLF	MDD	FA	↓	250	281	Case-control	Acute MDD vs remitted MDD. [41]
ATR	MDD	FA	↓	-	-	Meta 84 studies	[40]
PTR	MDD	FA	↓	-	-	Meta 84 studies	[40]
UNC	MDD	FA	↓	-	-	Meta 84 studies	[40]
CG	PTSD	FA	↓	322	335	Meta 14 studies	[28]
CST	PTSD	FA	↓	322	335	Meta 14 studies	[28]
GCC	PTSD	FA	↓	322	335	Meta 14 studies	[28]
PCR	PTSD	FA	↓	36	33	Case-control	[42]
TAP	PTSD	FA	↓	1377	1620	Meta 28 cohorts	[43]
ACR	SCZ	FA	↓	1377	1620	Meta 28 cohorts	[43]
ACR	SCZ	FA	↓	84	93	Case-control	[44]

ATR	SCZ	FA	↓	69369	236642	Case-control	UKB samples and PRS analysis. [45]
BCC	SCZ	FA	↓	696	1506	Case-control	[26]
BCC	SCZ	FA	↓	84	93	Case-control	[44]
Brainstem	SCZ	FA	↓	39	30	Case-control	Patient characteristic: never-treated first episode. [46]
CC	SCZ	FA	↓	696	1506	Case-control	[26]
CC	SCZ	FA	↓	1543	1612	Case-control	[29]
CC	SCZ	FA	↓	39	30	Case-control	Patient characteristic: never-treated first episode. [46]
CG	SCZ	FA	↓	1543	983	Case-control	The control was BD patients. [29]
CG	SCZ	FA	↓	39	30	Case-control	Patient characteristic: never-treated first episode. [46]
CGC	SCZ	FA	↓	600	492	Cross-sectional, from 13 different sites	Multiple illness stages analysis Stable trajectory [47]
CGH	SCZ	FA	↓	600	492	Cross-sectional, from 13 different sites	Multiple illness stages analysis Stable trajectory [47]
CGC	SCZ	FA	↓	696	1506	Case-control	[26]
EC	SCZ	FA	↓	49	123	Case-control	[48]
Forceps major	SCZ	FA	↓	600	492	Cross-sectional, from 13 different sites	Multiple illness stages analysis Stable trajectory [47]
Forceps minor	SCZ	FA	↓	600	492	Cross-sectional, from 13 different sites	Multiple illness stages analysis Stable trajectory [47]
Forceps minor	SCZ	FA	↓	69369	236642	Case-control	UKB samples and PRS analysis. [45]
FX	SCZ	FA	↓	696	1506	Case-control	[26]
GCC	SCZ	FA	↓	84	93	Case-control	[44]
IC	SCZ	FA	↓	39	30	Case-control	Patient characteristic: never-treated first episode. [46]
IFO	SCZ	FA	↓	600	492	Cross-sectional, from 13 different sites	Multiple illness stages analysis Stable trajectory [47]
ILF	SCZ	FA	↓	600	492	Cross-sectional, from 13 different sites	Multiple illness stages analysis Stable trajectory [47]
SCR	SCZ	FA	↓	84	93	Case-control	[44]

SLF	SCZ	FA	↓	600	492	Cross-sectional, from 13 different sites	Multiple illness stages analysis Stable trajectory [47]
STR	SCZ	FA	↓	69369	236642	Case-control	UKB samples and PRS analysis. [45]
UNC	SCZ	FA	↓	600	492	Cross-sectional, from 13 different sites	Multiple illness stages analysis Stable trajectory [47]
Whole brain	SCZ	FA	↓	518	452	Cross-sectional, 13 independent and international sites	[49]
UNC	ADHD	FA	-	-	-	Meta 13 studies	[30]
CC	BD	FA AD MD	-	-	-	Descriptive review 10 studies	[50]
IFO	ASD	MD	↑	147	162	Meta 6 studies	[35]
CC	ADHD	MD	↑	55	50	Case-control	[7]
ALIC	ASD	MD	↑	26	26	Cross-sectional	[33]
ATR	ASD	MD	↑	26	26	Cross-sectional	[33]
BCC	ASD	MD	↑	26	26	Cross-sectional	[33]
CC	ASD	MD	↑	69	50		[7]
Arcuate fasciculus/SLF	ASD	MD	↑	245	242	Meta 12 studies	[35]
Forceps major	ASD	MD	↑	26	26	Cross-sectional	[33]
Forceps minor	ASD	MD	↑	26	26	Cross-sectional	[33]
IFO	ASD	MD	↑	26	26	Cross-sectional	[33]
ILF	ASD	MD	↑	-	-	Meta 6 studies	[35]
ILF	ASD	MD	↑	26	26	Cross-sectional	[33]
PLIC	ASD	MD	↑	26	26	Cross-sectional	[33]
SCC	ASD	MD	↑	26	26	Cross-sectional	[33]
SLF	ASD	MD	↑	26	26	Cross-sectional	[33]
UNC	ASD	MD	↑	164	179	Meta 7 studies	[35]
UNC	ASD	MD	↑	26	26	Cross-sectional	[33]

BCC	BD	MD	↑	211	1506	Case-control	[26]
FX	BD	MD	↑	211	1506	Case-control	[26]
SLF	MDD	MD	↑	250	281	Case-control	Acute MDD vs remitted MDD. [41]
BCG	SCZ	MD	↑	696	1506	Case-control	[26]
CC	SCZ	MD	↑	696	1506	Case-control	[26]
CGC	SCZ	MD	↑	69369	236642	Case-control	UKB samples and PRS analysis. [45]
Forceps minor	SCZ	MD	↑	69369	236642	Case-control	UKB samples and PRS analysis. [45]
FX	SCZ	MD	↑	696	1506	Case-control	[26]
UNC	SCZ	MD	↑	696	1506	Case-control	[26]
UNF	SCZ	MD	↑	69369	236642	Case-control	UKB samples and PRS analysis. [45]
TR	ADHD	MD	↓	50	50	Sibling design, RCT	Only showed significant difference in ADHD vs siblings. [2]
CC	ADHD	RD	↑	55	50	Case-control	[7]
ALIC	ASD	RD	↑	26	26	Cross-sectional	[33]
ATR	ASD	RD	↑	26	26	Cross-sectional	[33]
BCC	ASD	RD	↑	26	26	Cross-sectional	[33]
CC	ASD	RD	↑	69	50	Case-control	[7]
Forceps major	ASD	RD	↑	26	26	Cross-sectional	[33]
Forceps minor	ASD	RD	↑	26	26	Cross-sectional	[33]
IFO	ASD	RD	↑	26	26	Cross-sectional	[33]
ILF	ASD	RD	↑	26	26	Cross-sectional	[33]
SCC	ASD	RD	↑	26	26	Cross-sectional	[33]
SLF	ASD	RD	↑	26	26	Cross-sectional	[33]
UNC	ASD	RD	↑	26	26	Cross-sectional	[33]
CC	BD	RD	↑	462	511	Meta<br>15 studies	[37]
CST	BD	RD	↑	36	38	Case-control	[27]
EC	BD	RD	↑	49	123	Case-control	[48]

Fronto-orbito-polar tracts	BD	RD	↑	462	511	Case-control	[37]
GCC	BD	RD	↑	36	38	Case-control	[27]
SLF	BD	RD	↑	-	-	Meta 15 studies	[37]
ATR	BD	RD	↑	-	-	Meta 15 studies	[37]
SLF	MDD	RD	↑	250	281	Case-control	Acute MDD vs remitted MDD. [41]
Tapetum	PTSD	RD	↑	1377	1620	Meta 28 cohorts	[43]
ACR	SCZ	RD	↑	696	1506	Case-control	[26]
BCC	SCZ	RD	↑	696	1506	Case-control	[26]
CC	SCZ	RD	↑	696	1506	Case-control	[26]
CGC	SCZ	RD	↑	696	1506	Case-control	[26]
EC	SCZ	RD	↑	49	123	Case-control	[48]
FX	SCZ	RD	↑	696	1506	Case-control	[26]
UNC	SCZ	RD	↑	696	1506	Case-control	[26]
Fronto-striatal tract	ADHD	RD	↓	50	50	Sibling design, RCT	Only showed significant difference in ADHD vs siblings. [2]
TR	ADHD	RD	↓	50	50	Sibling design, RCT	Only showed significant difference in ADHD vs siblings. [2]
FX	BD	RD	↓	211	1506	Case-control	[26]

Abbreviations: AC: anterior commissure; ACR: anterior corona radiata; AD: axial diusivities; ALIC: anterior limb of internal capsule; ASD: Autism disorderTS: Tourette syndromeADHD: Attention deficit hyperactivity disorder; ATR: anterior thalamic radiation; AUD: alcohol use disorder; CUD: cannabis use disorderOUD: opioid use disorder; BCC: body of corpus callosum; CC: corpus callosum; CGC: cingulum connecting to cingulate gyrus; CGH: cingulum connecting to hippocampus; CP: cerebellar peduncle; CR: corona radiata; CST: corticospinal tract; EC: external capsule; FA: fractional anisotropy, white matter integrity; FX/ST: fornix and stria terminalis; FX: Fornix (column and body of fornix); GCC: genu of corpus callosum; IC: internal capsule; ICP: inferior cerebellar peduncle; IFO/ILF: inferior fronto-occipital fasciculus/inferior longitudinal fasciculus; IFO/UNC: inferior fronto-occipital fasciculus/uncinate fasciculus; ILF: inferior longitudinal fasciculus; MCP: medial cerebellar peduncle; MD: mean diusivities, cell damage; MDD: major depression; PTSD: post-traumatic stress disorder; ML: medial lemniscus; MLF: medial longitudinal fasciculus; MO: mode of anisotropy; PCR: posterior corona radiata; PLIC: posterior limb of internal capsule; PRS: polygenic risk score; PTR: posterior thalamic radiation (include optic radiation); PTR: Posterior thalamic radiation (include optic radiation); RCT: randomized controlled trail; RD: radial diusivities, myelin integrity; RLIC: retrolenticular part of internal capsule; SCC: splenium of corpus callosum; SCP: superior cerebellar peduncle; SCR: superior corona radiata; SCZ: schizophrenia; BD: bipolar disorder; PD: panic disorder; SFO: superior fronto-occipital fasciculus; SLF:

superior longitudinal fasciculus; SS: Sagittal stratum; STR: superior thalamic radiation; TAP: tapetum TR: thalamic radiation; UKB: UK biobank; UNC: uncinate fasciculus.

Table S2. STROBE-MR checklist of recommended items to address in reports of Mendelian randomization studies [51,52]

Item No.	Section		Checklist item	Location addressed
1	TITLE and ABSTRACT		Indicate Mendelian randomization (MR) as the study's design in the title and/or the abstract if that is a main purpose of the study	Title and abstract.
INTRODUCTION				
2	Background		Explain the scientific background and rationale for the reported study. What is the exposure? Is a potential causal association between exposure and outcome plausible? Justify why MR is a helpful method to address the study question	Introduction.
3	Objectives		State specific objectives clearly, including pre-specified causal hypotheses (if any). State that MR is a method that, under specific assumptions, intends to estimate causal effects	Introduction.
METHODS				
4	Study design and data sources		Present key elements of the study design early in the article. Consider including a table listing sources of data for all phases of the study. For each data source contributing to the analysis, describe the following:	a) Introduction, Methods, Figure 1, Supplementary Data 1 - 3.<br>b) Methods, Supplementary Data 1 - 2.<br>c) Methods.<br>d) Methods.<br>e) Methods.
		a)	Setting: Describe the study design and the underlying population, if possible. Describe the setting, locations, and relevant dates, including periods of recruitment, exposure, follow-up, and data collection, when available.	
		b)	Participants: Give the eligibility criteria, and the sources and methods of selection of participants. Report the sample size, and whether any power or sample size calculations were carried out prior to the main analysis	
		c)	Describe measurement, quality control and selection of genetic variants	
		d)	For each exposure, outcome, and other relevant variables, describe methods of assessment and diagnostic criteria for diseases	
		e)	Provide details of ethics committee approval and participant informed consent, if relevant	
5	Assumptions		Explicitly state the three core IV assumptions for the main analysis (relevance, independence and exclusion restriction) as well assumptions for any additional or sensitivity analysis	Figure 1, Methods.
6	Statistical methods: main analysis		Describe statistical methods and statistics used	a) Introduction, Methods, Figure 1, Supplementary Data 1 - 2.<br>b) Methods, Supplementary Data 1 - 2.<br>c) Methods.<br>d) Methods.
		a)	Describe how quantitative variables were handled in the analyses (i.e., scale, units, model)	
		b)	Describe how genetic variants were handled in the analyses and, if applicable, how their weights were selected	

		c)	Describe the MR estimator (e.g. two-stage least squares, Wald ratio) and related statistics. Detail the included covariates and, in case of two-sample MR, whether the same covariate set was used for adjustment in the two samples	e) Methods.
		d)	Explain how missing data were addressed	
		e)	If applicable, indicate how multiple testing was addressed	
7	Assessment of assumptions		Describe any methods or prior knowledge used to assess the assumptions or justify their validity	Methods, Figure 1.
8	Sensitivity analyses and additional analyses		Describe any sensitivity analyses or additional analyses performed (e.g. comparison of effect estimates from different approaches, independent replication, bias analytic techniques, validation of instruments, simulations)	Methods, Figure 1.
9	Software and pre-registration			a) Methods.<br>b) The research protocol and information are not registered.
		a)	Name statistical software and package(s), including version and settings used	
		b)	State whether the study protocol and details were pre-registered (as well as when and where)	
RESULTS				
10	Descriptive data			a) Not applicable, since, in this study, we used genome-wide summary statistics from previously published genome-wide association studies.<br>b) Results, Methods, Supplementary Data 1 - 2.<br>c) Not applicable, since, in this study, we used genome-wide summary statistics from previously published genome-wide association studies.<br>d) Results, Supplementary Data 2 - 3.
		a)	Report the numbers of individuals at each stage of included studies and reasons for exclusion. Consider use of a flow diagram	
		b)	Report summary statistics for phenotypic exposure(s), outcome(s), and other relevant variables (e.g. means, SDs, proportions)	
		c)	If the data sources include meta-analyses of previous studies, provide the assessments of heterogeneity across these studies	
		d)	For two-sample MR:<br>i. Provide justification of the similarity of the genetic variant-exposure associations between the exposure and outcome samples<br>ii. Provide information on the number of individuals who overlap between the exposure and outcome studies	
11	Main results			a) Not applicable, since in this study we used genome-wide summary statistics from previously published genome-wide association studies. No individual level data available.<br>b) Results, Supplementary Data 2 - 3.
		a)	Report the associations between genetic variant and exposure, and between genetic variant and outcome, preferably on an interpretable scale	
		b)	Report MR estimates of the association between exposure and outcome, and the measures of uncertainty	

			from the MR analysis, on an interpretable scale, such as odds ratio or relative risk per SD difference	c) Not applicable.<br>d) Figures 2 - 3 and Figures S1 - S25.
		c)	If relevant, consider translating estimates of relative risk into absolute risk for a meaningful time period	
		d)	Consider plots to visualize results (e.g. forest plot, scatterplot of associations between genetic variants and outcome versus between genetic variants and exposure)	
12	Assessment of assumptions			a) Methods.<br>b) Results, Supplementary Data 13 - 14.
		a)	Report the assessment of the validity of the assumptions	
		b)	Report any additional statistics (e.g., assessments of heterogeneity across genetic variants, such as I^2, Q statistic or E-value)	
13	Sensitivity analyses and additional analyses			a) Results, Supplementary Data 13 - 20.<br>b) Results, Supplementary Data 13 - 20.<br>c) Results, Supplementary Data 13 - 20.<br>d) Introduction, Results, Discussion, Table S1.<br>e) Figures 2 - 4 and Figures S1 - S25.
		a)	Report any sensitivity analyses to assess the robustness of the main results to violations of the assumptions	
		b)	Report results from other sensitivity analyses or additional analyses	
		c)	Report any assessment of direction of causal association (e.g., bidirectional MR)	
		d)	When relevant, report and compare with estimates from non-MR analyses	
		e)	Consider additional plots to visualize results (e.g., leave-one-out analyses)	
DISCUSSION				
14	Key results		Summarize key results with reference to study objectives	Discussion.
15	Limitations		Discuss limitations of the study, taking into account the validity of the IV assumptions, other sources of potential bias, and imprecision. Discuss both direction and magnitude of any potential bias and any efforts to address them	Methods, Discussion.
16	Interpretation			a) Discussion.<br>b) Discussion, Supplementary Data 20.<br>c) Abstract, Introduction, Discussion.
		a)	Meaning: Give a cautious overall interpretation of results in the context of their limitations and in comparison with other studies	
		b)	Mechanism: Discuss underlying biological mechanisms that could drive a potential causal association between the investigated exposure and the outcome, and whether the gene-environment equivalence assumption is reasonable. Use causal language carefully, clarifying that IV estimates may provide causal effects only under certain assumptions	

		c) Clinical relevance: Discuss whether the results have clinical or public policy relevance, and to what extent they inform effect sizes of possible interventions	
17	Generalizability	Discuss the generalizability of the study results (a) to other populations, (b) across other exposure periods/timings, and (c) across other levels of exposure	a) Discussion.<br>b) Discussion.<br>c) Not applicable.
OTHER INFORMATION			
18	Funding	Describe sources of funding and the role of funders in the present study and, if applicable, sources of funding for the databases and original study or studies on which the present study is based	Acknowledgments.
19	Data and data sharing	Provide the data used to perform all analyses or report where and how the data can be accessed, and reference these sources in the article. Provide the statistical code needed to reproduce the results in the article, or report whether the code is publicly accessible and if so, where	Methods, Supplementary Data 1 - 11, Reference, Supplementary Materials and R codes.
20	Conflicts of Interest	All authors should declare all potential conflicts of interest	The authors declare that there is no conflict of interest.

This checklist is copyrighted by the Equator Network under the Creative Commons Attribution 3.0 Unported (CC BY 3.0) license.

References

1. Yang, A., *et al.* Longer screen time utilization is associated with the polygenic risk for Attention-deficit/hyperactivity disorder with mediation by brain white matter microstructure. *EBioMedicine* **80**, 104039 (2022).
2. Chiang, H.L., Hsu, Y.C., Shang, C.Y., Tseng, W.I. & Gau, S.S. White matter endophenotype candidates for ADHD: a diffusion imaging tractography study with sibling design. *Psychol Med* **50**, 1203-1213 (2020).
3. Versace, A., *et al.* White matter abnormalities associated with ADHD outcomes in adulthood. *Mol Psychiatry* **26**, 6655-6665 (2021).
4. Dziemian, S., Barańczuk-Turska, Z. & Langer, N. Association between attention-deficit/hyperactivity disorder symptom severity and white matter integrity moderated by in-scanner head motion. *Transl Psychiatry* **12**, 434 (2022).
5. Zhang, K., *et al.* The shared white matter developmental trajectory anomalies of attention-deficit/hyperactivity disorder and autism spectrum disorders: A meta-analysis of diffusion tensor imaging studies. *Prog Neuropsychopharmacol Biol Psychiatry* **124**, 110731 (2023).
6. Damatac, C.G., *et al.* White Matter Microstructure in Attention-Deficit/Hyperactivity Disorder: A Systematic Tractography Study in 654 Individuals. *Biol Psychiatry Cogn Neurosci Neuroimaging* **7**, 979-988 (2022).
7. Aoki, Y., *et al.* Association of White Matter Structure With Autism Spectrum Disorder and Attention-Deficit/Hyperactivity Disorder. *JAMA Psychiatry* **74**, 1120-1128 (2017).
8. Andrews, D.S., *et al.* A Longitudinal Study of White Matter Development in Relation to Changes in Autism Severity Across Early Childhood. *Biol Psychiatry* **89**, 424-432 (2021).
9. Hegarty, J.P., 2nd, *et al.* A Twin Study of Altered White Matter Heritability in Youth With Autism Spectrum Disorder. *J Am Acad Child Adolesc Psychiatry* **63**, 65-79 (2024).
10. Tura, A. & Goya-Maldonado, R. Brain connectivity in major depressive disorder: a precision component of treatment modalities? *Transl Psychiatry* **13**, 196 (2023).
11. Ping, L., *et al.* Tryptophan hydroxylase-2 polymorphism is associated with white matter integrity in first-episode, medication-naïve major depressive disorder patients. *Psychiatry Res Neuroimaging* **286**, 4-10 (2019).
12. Flinkenflügel, K., *et al.* Negative Stressful Life Events and Social Support Are Associated With White Matter Integrity in Depressed Patients and Healthy Control Participants: A Diffusion Tensor Imaging Study. *Biol Psychiatry* **94**, 650-660 (2023).
13. Hu, C., *et al.* Microstructural abnormalities of white matter in the cingulum bundle of adolescents with major depression and non-suicidal self-injury. *Psychol Med* **54**, 1113-1121 (2024).
14. Wang, L., *et al.* Associations between the kynurenine pathway and the brain in patients with major depressive disorder-A systematic review of neuroimaging studies. *Prog Neuropsychopharmacol Biol Psychiatry* **121**, 110675 (2023).
15. Comai, S., *et al.* Selective association of cytokine levels and kynurenine/tryptophan ratio with alterations in white matter microstructure in bipolar but not in unipolar depression. *Eur Neuropsychopharmacol* **55**, 96-109 (2022).
16. Delvecchio, G., Pigoni, A., Bauer, I.E., Soares, J.C. & Brambilla, P. Disease-discordant twin structural MRI studies on affective disorders. *Neurosci Biobehav Rev* **108**, 459-471 (2020).
17. Thiel, K., *et al.* Reduced fractional anisotropy in bipolar disorder v. major depressive disorder independent of current symptoms. *Psychol Med* **53**, 4592-4602 (2023).
18. Thiel, K., *et al.* White and gray matter alterations in bipolar I and bipolar II disorder subtypes compared with healthy controls - exploring associations with disease course and polygenic risk. *Neuropsychopharmacology* **49**, 814-823 (2024).
19. Caruana, G.F., Carruthers, S.P., Berk, M., Rossell, S.L. & Van Rheenen, T.E. To what extent does white matter map to cognition in bipolar disorder? A systematic review of the evidence. *Prog Neuropsychopharmacol Biol Psychiatry* **128**, 110868 (2024).
20. Stein, F., *et al.* Brain Structural Network Connectivity of Formal Thought Disorder Dimensions in Affective and Psychotic Disorders. *Biol Psychiatry* **95**, 629-638 (2024).

21. Seitz-Holland, J., *et al.* Shared and distinct white matter abnormalities in adolescent-onset schizophrenia and adolescent-onset psychotic bipolar disorder. *Psychol Med* **53**, 4707-4719 (2023).
22. Aronica, R., Enrico, P., Squarcina, L., Brambilla, P. & Delvecchio, G. Association between Diffusion Tensor Imaging, inflammation and immunological alterations in unipolar and bipolar depression: A review. *Neurosci Biobehav Rev* **143**, 104922 (2022).
23. Goldwaser, E.L., *et al.* White matter in prolonged glucocorticoid response to psychological stress in schizophrenia. *Neuropsychopharmacology* **46**, 2312-2319 (2021).
24. Wong, S.A., *et al.* Internal capsule microstructure mediates the relationship between childhood maltreatment and PTSD following adulthood trauma exposure. *Mol Psychiatry* **28**, 5140-5149 (2023).
25. Zhang, F., *et al.* Genetic evidence suggests posttraumatic stress disorder as a subtype of major depressive disorder. *J Clin Invest* **132**(2022).
26. Koshiyama, D., *et al.* White matter microstructural alterations across four major psychiatric disorders: mega-analysis study in 2937 individuals. *Mol Psychiatry* **25**, 883-895 (2020).
27. Linke, J.O., *et al.* White Matter Microstructure in Pediatric Bipolar Disorder and Disruptive Mood Dysregulation Disorder. *J Am Acad Child Adolesc Psychiatry* **59**, 1135-1145 (2020).
28. Ju, Y., *et al.* White matter microstructural alterations in posttraumatic stress disorder: An ROI and whole-brain based meta-analysis. *J Affect Disord* **266**, 655-670 (2020).
29. Zhao, G., *et al.* A Comparative Multimodal Meta-analysis of Anisotropy and Volume Abnormalities in White Matter in People Suffering From Bipolar Disorder or Schizophrenia. *Schizophr Bull* **48**, 69-79 (2022).
30. Parlatini, V., *et al.* White matter alterations in Attention-Deficit/Hyperactivity Disorder (ADHD): a systematic review of 129 diffusion imaging studies with meta-analysis. *Mol Psychiatry* **28**, 4098-4123 (2023).
31. Sudre, G., *et al.* A Mega-analytic Study of White Matter Microstructural Differences Across 5 Cohorts of Youths With Attention-Deficit/Hyperactivity Disorder. *Biol Psychiatry* **94**, 18-28 (2023).
32. Nagel, B.J., *et al.* Altered white matter microstructure in children with attention-deficit/hyperactivity disorder. *J Am Acad Child Adolesc Psychiatry* **50**, 283-292 (2011).
33. Andica, C., *et al.* Neurite orientation dispersion and density imaging reveals white matter microstructural alterations in adults with autism. *Mol Autism* **12**, 48 (2021).
34. Wilkes, B.J., *et al.* Cortico-basal ganglia white matter microstructure is linked to restricted repetitive behavior in autism spectrum disorder. *Mol Autism* **15**, 6 (2024).
35. Li, M., Wang, Y., Tachibana, M., Rahman, S. & Kagitani-Shimono, K. Atypical structural connectivity of language networks in autism spectrum disorder: A meta-analysis of diffusion tensor imaging studies. *Autism Res* **15**, 1585-1602 (2022).
36. Jiang, X., *et al.* Association of polygenic risk for bipolar disorder with grey matter structure and white matter integrity in youth. *Transl Psychiatry* **13**, 322 (2023).
37. Hu, R., Stavish, C., Leibenluft, E. & Linke, J.O. White Matter Microstructure in Individuals With and At Risk for Bipolar Disorder: Evidence for an Endophenotype From a Voxel-Based Meta-analysis. *Biol Psychiatry Cogn Neurosci Neuroimaging* **5**, 1104-1113 (2020).
38. Favre, P., *et al.* Widespread white matter microstructural abnormalities in bipolar disorder: evidence from mega- and meta-analyses across 3033 individuals. *Neuropsychopharmacology* **44**, 2285-2293 (2019).
39. Nothdurfter, D., Jawinski, P. & Markett, S. White Matter Tract Integrity Is Reduced in Depression and in Individuals With Genetic Liability to Depression. *Biol Psychiatry* **95**, 1063-1071 (2024).
40. Luttenbacher, I., *et al.* Transdiagnostic role of glutamate and white matter damage in neuropsychiatric disorders: A Systematic Review. *J Psychiatr Res* **147**, 324-348 (2022).

41. Meinert, S., *et al.* White matter fiber microstructure is associated with prior hospitalizations rather than acute symptomatology in major depressive disorder. *Psychol Med* **52**, 1166-1174 (2022).
42. Korgaonkar, M.S., *et al.* White matter anisotropy and response to cognitive behavior therapy for posttraumatic stress disorder. *Transl Psychiatry* **11**, 14 (2021).
43. Dennis, E.L., *et al.* Altered white matter microstructural organization in posttraumatic stress disorder across 3047 adults: results from the PGC-ENIGMA PTSD consortium. *Mol Psychiatry* **26**, 4315-4330 (2021).
44. Su, W., *et al.* Effects of polygenic risk of schizophrenia on interhemispheric callosal white matter integrity and frontotemporal functional connectivity in first-episode schizophrenia. *Psychol Med* **53**, 2868-2877 (2023).
45. Stauffer, E.M., *et al.* Grey and white matter microstructure is associated with polygenic risk for schizophrenia. *Mol Psychiatry* **26**, 7709-7718 (2021).
46. Yang, M., Gao, S. & Zhang, X. Cognitive deficits and white matter abnormalities in never-treated first-episode schizophrenia. *Transl Psychiatry* **10**, 368 (2020).
47. Cetin-Karayumak, S., *et al.* White matter abnormalities across the lifespan of schizophrenia: a harmonized multi-site diffusion MRI study. *Mol Psychiatry* **25**, 3208-3219 (2020).
48. Joo, S.W., *et al.* Shared and distinct white matter abnormalities in schizophrenia and bipolar disorder. *Prog Neuropsychopharmacol Biol Psychiatry* **108**, 110175 (2021).
49. Seitz-Holland, J., *et al.* Cognitive deficits, clinical variables, and white matter microstructure in schizophrenia: a multisite harmonization study. *Mol Psychiatry* **27**, 3719-3730 (2022).
50. Videtta, G., *et al.* White matter modifications of corpus callosum in bipolar disorder: A DTI tractography review. *J Affect Disord* **338**, 220-227 (2023).
51. Burgess, S., *et al.* Guidelines for performing Mendelian randomization investigations: update for summer 2023. *Wellcome Open Res* **4**, 186 (2019).
52. Skrivankova, V.W., *et al.* Strengthening the Reporting of Observational Studies in Epidemiology Using Mendelian Randomization: The STROBE-MR Statement. *Jama* **326**, 1614-1621 (2021).

Legends for Supplementary Data 1 to Supplementary Data 23

Supplementary Data 1. GWAS summary-level data of white matter microstructures.

Supplementary Data 2. GWAS summary-level data of psychiatric disorders.

Supplementary Data 3. IVs in forward MR (before confounder selection)

Supplementary Data 4. IVs in reverse MR.

Supplementary Data 5. IVs associated gwas traits in opengwas in forward MR

Supplementary Data 6. IVs associated gwas traits in opengwas in reverse MR

Supplementary Data 7. Confounders' selection of IVs

Supplementary Data 8. IVs in forward MR without confounders

Supplementary Data 9. Outliers detected by MR-PRESSO in forward MR analyses.

Supplementary Data 10. Outliers detected by MR-PRESSO in reverse MR analyses.

Supplementary Data 11. Information of IVs for all exposure-outcome pairs in forward MR analyses.

Supplementary Data 12. Information of IVs for all exposure-outcome pairs in reverse MR analyses.

Supplementary Data 13. Forward MR analysis results.

Supplementary Data 14. Rreverse MR analysis results.

Supplementary Data 15. Forward MR analysis results (before confounder fixing).

Supplementary Data 16. Comparing the IVW results before and after confounder filtering.

Supplementary Data 17. Comparing the IVW results before and after outlier filtering.

Supplementary Data 18. Significant forward and reverse MR results after adjusting and pleiotropy tests

Supplementary Data 19. Multiple comparison of significant MR results.

Supplementary Data 20. The summary and interpretation of significant MR results (with MRlap analysis).

Supplementary Data 21. MVMR analysis results.

Supplementary Data 22. Replication MR of BD, AUD, SCZ and ADHD in FinnGen R11

Supplementary Data 23. Significant MR results both in GWAS summary-level data(FDR_P < 5e-2) and replication data (P < 5e-2)
