# Supplementary material for: Unveiling causal relationship between white matter tracts and psychiatric disorders
Source: Commun Biol. 2025 Aug 14;8:1221. doi: 10.1038/s42003-025-08669-6 (PMC12354733; doi:10.1038/s42003-025-08669-6)
Supplement: Supplementary file 2 — Description of Additional Supplementary Files [file 42003_2025_8669_MOESM2_ESM.pdf]

Description of Additional Supplementary Files

File name: Supplementary Data 1

Description: GWAS summary-level data of white matter microstructures.

File name: Supplementary Data 2

Description: GWAS summary-level data of psychiatric disorders.

File name: Supplementary Data 3

Description: IVs in forward MR (before confounder selection)

File name: Supplementary Data 4

Description: IVs in reverse MR.

File name: Supplementary Data 5

Description: IVs associated gwas traits in opengwas in forward MR

File name: Supplementary Data 6

Description: IVs associated gwas traits in opengwas in reverse MR

File name: Supplementary Data 7

Description: Confounders' selection of IVs

File name: Supplementary Data 8

Description: IVs in forward MR without confounders

File name: Supplementary Data 9

Description: Outliers detected by MR-PRESSO in forward MR analyses

File name: Supplementary Data 10

Description: Outliers detected by MR-PRESSO in reverse MR analyses

File name: Supplementary Data 11

Description: Information of IVs for all exposure-outcome pairs in forward MR analyses

File name: Supplementary Data 12

Description: Information of IVs for all exposure-outcome pairs in reverse MR analyses

File name: Supplementary Data 13

Description: Forward MR analysis results

File name: Supplementary Data 14

Description: Rreverse MR analysis results

File name: Supplementary Data 15

Description: Forward MR analysis results (before confounder fixing)

File name: Supplementary Data 16

Description: Comparing the IVW results before and after confounder filtering

File name: Supplementary Data 17

Description: Comparing the IVW results before and after outlier filtering.

File name: Supplementary Data 18

Description: Significant forward and reverse MR results after adjusting and pleiotropy tests

File name: Supplementary Data 19

Description: Multiple comparison of significant MR results.

File name: Supplementary Data 20

Description: The summary and interpretation of significant MR results (with MRlap analysis)

File name: Supplementary Data 21

Description: MVMR analysis results

File name: Supplementary Data 22

Description: Replication MR of BD, AUD, SCZ and ADHD in FinnGen R11

File name: Supplementary Data 23

Description: Significant MR results both in GWAS summary-level data(FDR_P < 5e-2) and replication data (P < 5e-2)
